# NF-Y Recruits Ash2L to Impart H3K4 Trimethylation on CCAAT Promoters

**DOI:** 10.1371/journal.pone.0017220

**Published:** 2011-03-21

**Authors:** Andrea Fossati, Diletta Dolfini, Giacomo Donati, Roberto Mantovani

**Affiliations:** Dipartimento di Scienze Biomolecolari e Biotecnologie, Università degli Studi di Milano, Milano, Italy; Ludwig-Maximilians-Universität München, Germany

## Abstract

**Background:**

Different histone post-translational modifications (PTMs) are crucial in the regulation of chromatin, including methylations of H3 at Lysine 4 by the MLL complex. A relevant issue is how this is causally correlated to the binding of specific transcription factors (TFs) in regulatory regions. NF-Y is a TF that regulates 30% of mammalian promoters containing the widespread CCAAT element. We and others established that the presence of H3K4me3 is dependent upon the binding of NF-Y. Here, we investigate the mechanisms of H3K4me3 deposition by NF-Y.

**Methods:**

We employed Chromatin Immunoprecipitation in cells in which Ash2L and NF-Y subunits were knocked down by RNAi, to monitor the presence of histones PTMs and components of the MLL complex. We performed gene expression profiling of Ash2L-knocked down cells and analyzed the regulated genes. We performed ChIPs in leukemic cells in which MLL1 is devoid of the methyltransferase domain and fused to the AF4 gene.

**Results:**

Knock down of the Ash2L subunit of MLL leads to a decrease in global H3K4me3 with a concomitant increase in H3K79me2. Knock down of NF-Y subunits prevents promoter association of Ash2L, but not MLL1, nor WDR5, and H3K4me3 drops dramatically. Endogenous NF-Y and Ash2L specifically interact *in vivo*. Analysis of the promoters of Ash2L regulated genes, identified by transcriptional profiling, suggests that a handful TF binding sites are moderately enriched, among which the CCAAT box. Finally, leukemic cells carrying the MLL-AF4 translocation show a decrease of H3K4me3, absence of Ash2L and increase in H3K79me2, while NF-Y binding was not significantly affected.

**Conclusions:**

Three types of conclusions are reached: (i) H3K4 methylation is not absolutely required for NF-Y promoter association. (ii) NF-Y acts upstream of H3K4me3 deposition by recruiting Ash2L. (iii) There is a general cross-talk between H3K4me3 and H3K79me2 which is independent from the presence of MLL oncogenic fusions.

## Introduction

Histone post-translational modifications -PTMs- are marks of chromatin environments. Some of them are associated with accessible chromatin, others with heterochromatin, either constitutive or facultative [1]. Specifically, monoubiquitination of H2B at Lysine 120 –K123 in yeast- is one of the earliest events in the establishment of an active chromatin environment [2]. H3 methylations follow, on H3K4 and H3K79, in a hierarchy of events that leads to gene activation. Methylation of H3K4 is highly regulated [3] and generally present in active and poised promoters [4]. The major H3K4 Methyltransferase is MLL (ALL1), the human homologue of the *Drosophila* Trithorax, assembled in a complex that includes Menin, Ash2L, WDR5, RbBP5, DPY30 and HCFs [5]–[7]. The 4 MLL genes in humans, MLL1-4 contain a Set domain which mono-, di- and tri-methylates H3K4 [8]; proteins within the complex are important to impart substrate specificity [9]. Specifically, the complex is unable to tri-methylate H3K4 in the absence of Ash2L [6], [7]. Moreover, MLL1 is involved in chromosomal translocations with a large cohort of >50 *loci* in aggressive myeloid and lymphoid leukemias [10].

In general, a key question is how histone modifying complexes are selectively and timely recruited to promoters, and binding of sequence-specific transcription factors offers a convenient explanation [11]–[16]. NF-Y is a trimer composed of NF-YA, NF-YB and NF-YC [17], which regulates the CCAAT box, one of the most frequent and crucial promoter elements [18]. A connection between NF-Y binding and H3K4 methylations was initially noticed on the promoters of the ER-stress response genes, prior to induction [19], [20]. This was confirmed in genome-wide correlative ChIP on chip studies, since NF-Y and H3K4me3 locations overlapped significantly and correlated with expression [21]. In cause-effect experiments, we and others noticed a parallel decrease in NF-Y binding, H3K4me3, H3K79me2 and transcription using a dominant negative NF-YA mutant [22]–[24]. The reverse was not tested, namely whether H3K4me3 is important for NF-Y promoter association. This is a relevant point, since not all TFs are apparently equal in this regard: in a detailed correlative analysis, MYC binding was always associated with a specific context of histone marks, notably H3K4me3 and H3K79me2, but its removal –and comparison between myc^+/+^ and myc^−/−^ cells- left the H3K4 pattern intact at target sites, whereas H4 acetylations were substantially ablated. Therefore, it was concluded that these marks are required for MYC binding to E boxes [25]. In a previous study focusing on cell cycle regulated promoters in single nucleosome ChIP assays, we established that H3K4 di-methylation is unaffected by NF-Y binding, which is instead involved in the transition to mono- and tri-methylation upon gene activation. We then focused on H3K4me1: NF-Y promotes it by recruiting the CoREST-KDM1 H3K4me2 demethylase complex through contacts between NF-Y and CoREST [24]. Here, we report studies on the deposition of the H3K4me3 mark on CCAAT promoters. In particular, we first tested the hypothesis that H3K4me3 might be generally helpful in NF-Y promoter association, by eliminating Ash2L, the one subunit of the complex that is specifically required for the deposition of this mark.

## Results

### Knock down of Ash2L leads to decrease in H3K4me3, increase in H3K79me2 and selective reduction of NF-Y binding

**Figure 1:**
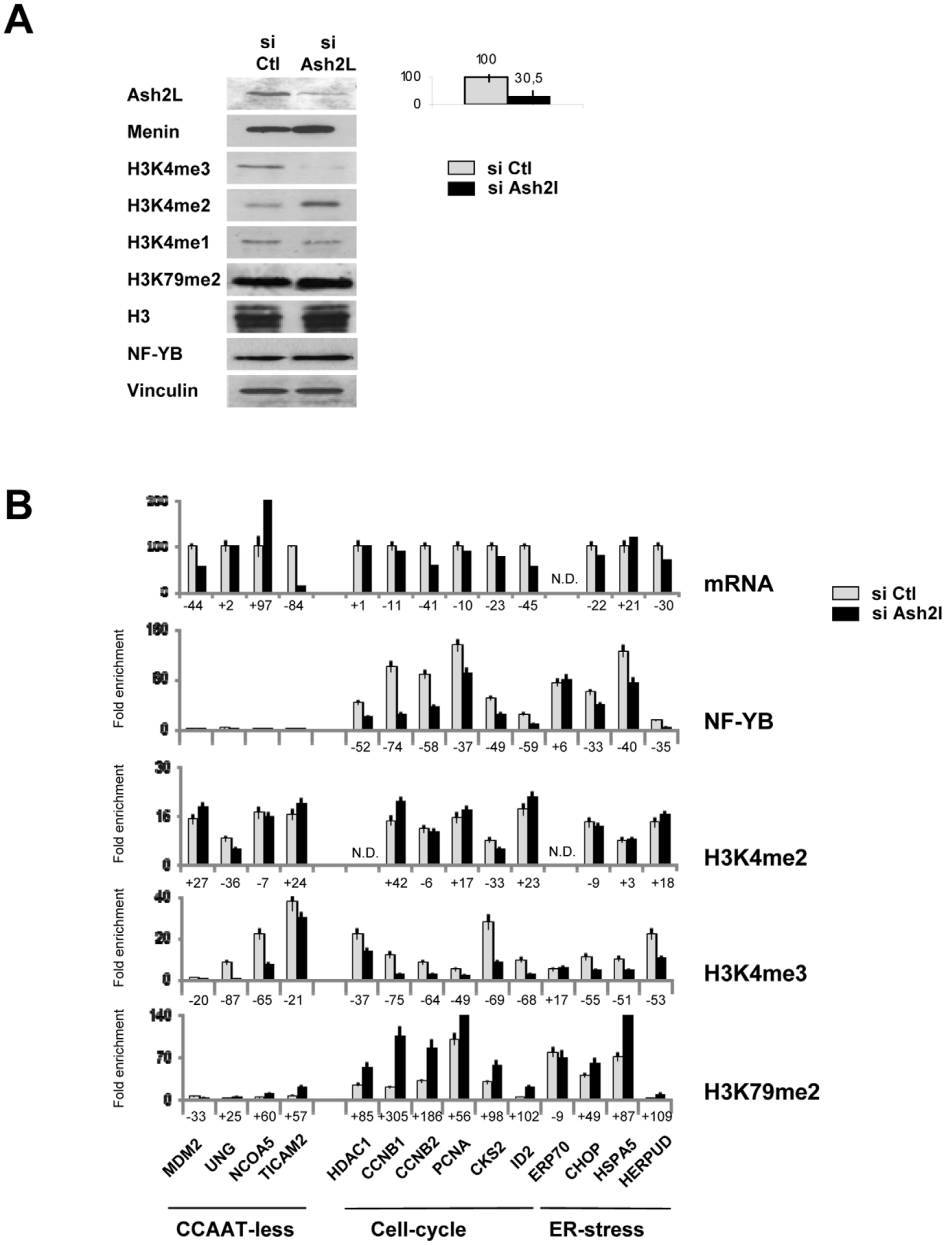
Effects of Ash2L knock down on H3K4me3, H3K79me2 and NF-Y binding. **A**. Knock down of Ash2L in HCT116 human cells. Western blot analysis of the indicated proteins and histone PTMs in cells transfected with scramble and Ash2L siRNAs. On the right, the levels of Ash2L protein inactivation, based on three independent experiments. **B**. In the upper Panel, mRNAs levels of the indicated genes in HCT116 cells transfected with scramble (Grey bars) or Ash2L siRNAs (Black bars) were assessed by qRT-PCR. In the lower Panels, Chromatin immunoprecipitation (ChIP) analysis was performed with the indicated antibodies (NF-YB, H3K4me2, H3K4me3, H3K79me2) in the same cells transfected with scramble or Ash2L siRNA. qPCR analysis was performed with primers centered in the core promoters of the indicated genes. CCAAT-less refers to genes with no CCAAT box in the promoters. Cell cycle and ER-stress are two categories of promoters with functionally important CCAAT boxes.

To study the role of H3K4me3 in NF-Y promoter association, we knocked down Ash2L by siRNA in HCT116 cells: Figure 1A shows that a substantial reduction of Ash2L –down to 30% of normal levels- could be achieved, while other subunits of MLL complexes, Menin and WDR5, were, if anything, increased (Fig. 1A). Global levels of H3K4 methylations were controlled by Western blot analysis: H3K4me3 reduction by Ash2L siRNA was matched by an increase of H3K4me2, while H3K4me1 was unchanged (Fig. 1A, Lower Panels). Next, we performed ChIP experiments with chromatin of HCT116 cells treated with control and Ash2L siRNAs, using antibodies against NF-Y, H3K4me3, H3K4me2, H3K79me2 and Ash2L; we analyzed a few promoters that are representatives of the different classes of CCAAT promoters -housekeeping, cell cycle and ER-stress- as well as of promoters devoid a of the CCAAT box -CCAAT-less- serving as controls. In parallel, we analyzed the transcriptional profile of the genes considered. Fig. 1B shows a two to four-fold decrease of H3K4me3 on most promoters, with the exception of TICAM2 and ERP70. Interestingly, H3K79me2 levels were concomitantly increased (2 to 3-fold): indeed, the greater the loss of H3K4me3, the higher the increase of H3K79me2. NF-Y binding was decreased on some promoters -ERP70, CHOP, PCNA but not abolished. In most other promoters, however, the decrease in NF-Y binding was marginal. We conclude that the presence of H3K4me3 moderately influences NF-Y binding only on some promoters, and that indirect removal of H3K4me3 by inactivation of Ash2L leads to a compensatory positive effect on H3K79me2.

### NF-Y recruits Ash2L on CCAAT-containing promoters

**Figure 2:**
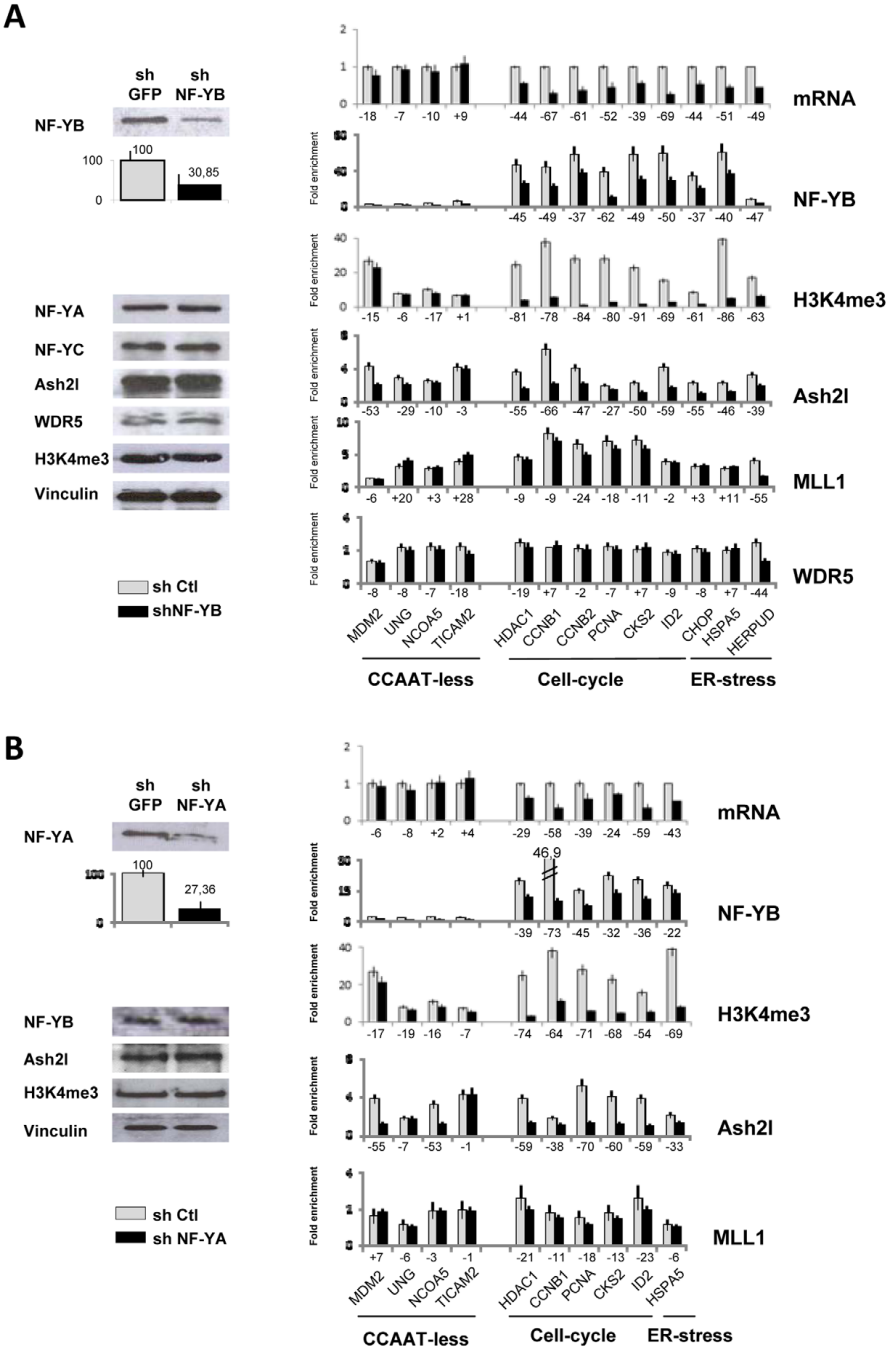
NF-Y recruits Ash2L on CCAAT-containing promoters. **A**. Knock down of the NF-YB subunit in HCT116 human cells by infection with control (GFP) or NF-YB shRNA producing Lentiviruses. Left Panels: Western blot analysis of the indicated proteins and histone PTMs, with a statistical evaluation of the degree of NF-YB protein knock down, measured based on three independent experiments. Right Panels: in the upper Panel, mRNAs levels of the indicated genes in HCT116 cells transfected with control GFP (Grey bars), or NF-YB shRNAs (Black bars) assessed by qRT-PCR. In the lower Panels, Chromatin immunoprecipitation (ChIP) analysis was performed with the indicated antibodies (NF-YB, H3K4me3, Ash2L, MLL1, WDR5) in the same cells. qPCR analysis was performed with primers centered in the core promoters of the genes. The same set of promoters of Figure 1 were analyzed. **B**. Same as A, except that cells were knocked down with shRNA for NF-YA.

Removal of the NF-Y trimer from cell cycle promoters by an NF-YA dominant negative mutant led to a substantial decrease of H3K4 tri-methylation [22]–[24], which is dependent from Ash2L, but not H3K4 di-methylation. One possibility is that NF-Y is important specifically for Ash2L recruitment. To establish this point, we decided to use an alternative system: knock down of the NF-YB (Fig. 2A) and of NF-YA (Fig. 2B) subunits in HCT116 cells by shRNA interference. Western blot analysis of NF-Y subunits confirmed the selective reduction of the respective NF-Y subunits: to 30% for NF-YB and 27% for NF-YA. Ash2L, as well as the global levels of H3K4me3 were not affected. ChIP assays on the NF-Y-dependent promoters analyzed in Fig. 1 showed a parallel reduction of NF-YB, H3K4me3 and Ash2L, whereas the presence of MLL1 and WDR5 was unaffected. On the control CCAAT-less UNG, COA5 and TICAM2 promoters, no NF-Y binding and robust Ash2L association was evident (Fig. 2A and 2B); MDM2, instead, showed a drop in Ash2L: a possible interpretation is that an important NF-Y site in a distant region impacts on promoter organization affecting H3K4me3.

**Figure 3:**
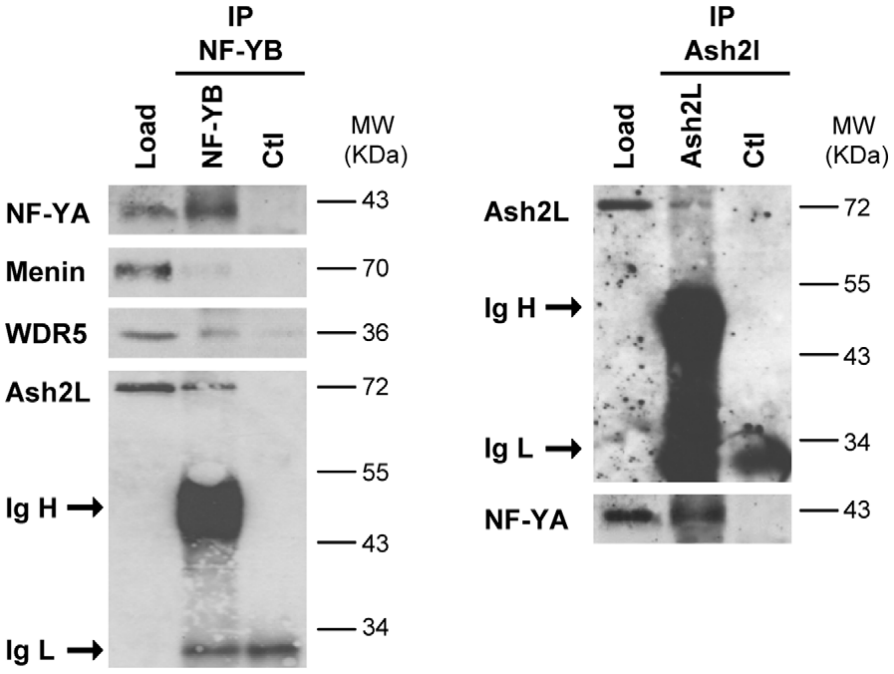
Direct interaction between NF-Y and Ash2L. Western blot analysis of immunprecipitations with anti-NF-YB (Left Panel) and anti-Ash2L (Right Panel) antibodies, using acid-extracted nuclear extracts of HCT116 cells. Control IPs with anti-Flag antibodies were run in parallel. The Load lane refers to the starting nuclear extract material. Menin and WDR5 were marginally immunoprcipitated with anti-NF-YB, whereas Ash2L recovery was robust. The heavy (IgH) and light (IgL) chains of the IP antibodies are indicated.

To ascertain whether there is a NF-Y-Ash2L interaction *in vivo*, we performed immunoprecipitations with NF-Y and Ash2L antibodies with HCT116 nuclear extracts, followed by Western blot analysis. Fig. 3 shows that Ash2L is immunoprecipitated with anti-NF-YB antibodies, whereas minimal levels of Menin and WDR5 were present in the bound fraction. The reciprocal was also true, as substantial amounts of NF-YA was in the Ash2L IP. Control IPs were negative for all proteins tested. Taken together, these data indicate that the recruitment of Ash2L is dependent upon NF-Y binding, that there is a NF-Y-Ash2L interaction *in vivo*, and that other subunits of the MLL complex are recruited independently from Ash2L.

**Figure 4:**
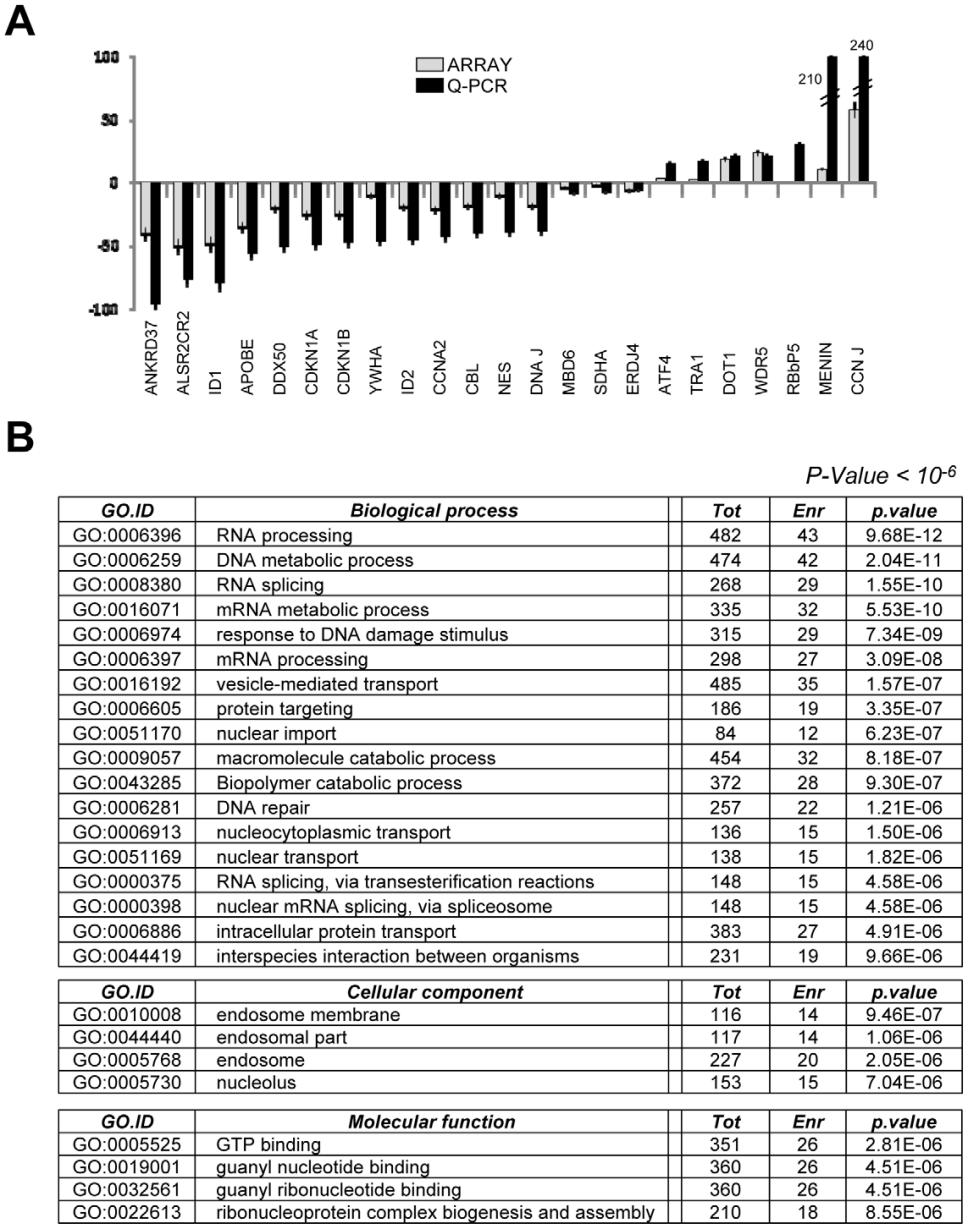
Validation of Ash2L profiling experiments. **A**. Validation of the profiling data for selected genes whose expression was activated or repressed by Ash2L inactivation, as in the experiments shown in Figure 1, was performed by qRT-PCR. **B**. Gene Ontology analysis of the profiling experiments of Ash2L-dependent genes.

To ascertain whether Ash2L is recruited preferentially on CCAAT promoters, we performed expression profiling of HCT116 cells after knock down of Ash2L by siRNA, with the protocol shown above in Fig. 1. By setting the threshold at 1.35, 477 genes were down-regulated and 175 up-regulated (Fig. S1). We validated by qRT-PCR 32 of these genes, and the adherence to the gene expression data was almost complete (Fig. 4A). The overall greater changes observed by qRT-PCR analysis suggest that additional genes below the threshold considered are affected by Ash2L interference. Analysis of Gene Ontology terms indicates that Ash2L targets different classes of genes, with *DNA* and *RNA Metabolism* being dominant in the Biological Process and Molecular Function categories (Fig. 4B). We then analyzed the regulated promoters to identify enriched Transcription Factor Binding Site -TFBS- (Figure S2): CCAAT was indeed at a top of a short list of sites, together with a few other, but the *p values* were relatively modest, an indication that there is no strong skewing toward a specific TFBS. We conclude that Ash2L recruitment on CCAAT promoters is NF-Y-dependent, but that the function of this MLL subunit is not restricted to CCAAT promoters.

### Ash2L promoters association is affected in Mixed Lineage Leukemia cells

**Figure 5:**
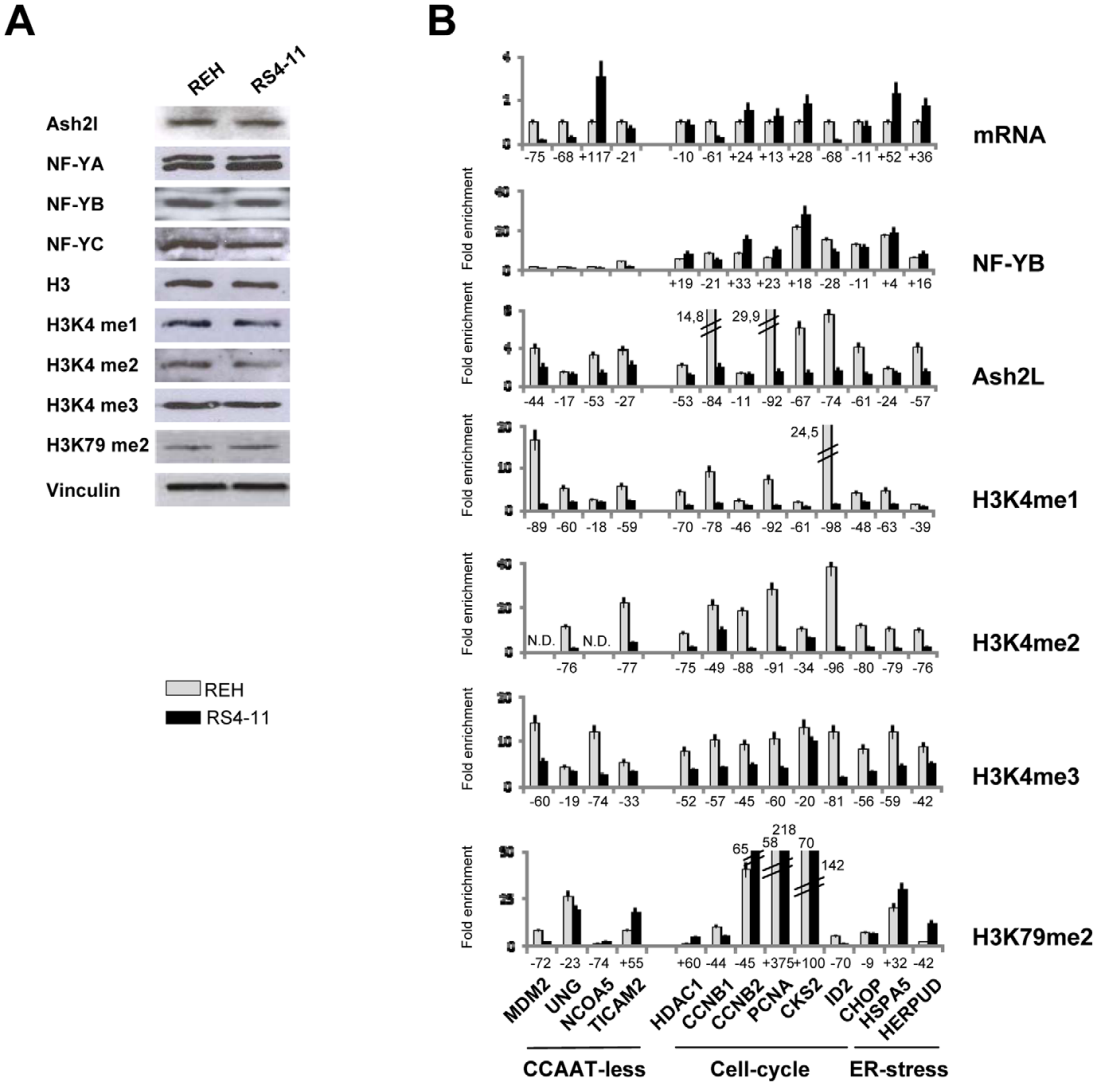
MLL cells lack Ash2L promoter association. **A**. Western blot analysis of the levels of the indicated proteins (Ash2L, NF-Y subunits, global H3, H3K4me1, H3K4me2, H3K4me3, H3K79me2, and the internal control Vinculin) in MLL1-AF4 rearranged cells RS4-11 and in non MLL1 rearranged REH leukemic cells. **B**. In the upper Panel, mRNAs levels of the indicated genes in REH (Black bars) and RS4-11 (Grey bars) assessed by qRT-PCR. In the lower Panels, Chromatin immunoprecipitation (ChIP) analysis was performed with the indicated antibodies (NF-YB, Ash2L, H3K4mei, H3K4me2, H3K4me3, H3K79me2) in the same cells. qPCR analysis was performed with primers centered in the core promoters of the genes. The same set of promoters of Figure 1 were analyzed.

The results shown above indicate that NF-Y lies upstream of Ash2L recruitment and H3K4me3 deposition. Previous data on cell cycle genes indicate that H3K4me2 is present independently from NF-Y, raising the possibility that this mark is actually required for NF-Y recruitment. To further evaluate this point, we used the cell line RS4-11, derived from a Mixed Lineage Leukemia, which contains a rearrangement of the MLL1 gene with AF4: as in other MLL1 fusions, it lacks the SET domain and it is therefore devoid of H3K4 methylating activity (10). Note that in MLL-AF10 regulated genes, this is compensated by higher levels of H3K79me2 [26]–[28], which is also under the control of NF-Y [22], [24]. Therefore, we first evaluated the global levels of the proteins and histone PTMs considered in this study in RS4-11, showing that they were largely similar to what is found in REH, a control leukemia cell line that harbours normal MLL1 alleles (Fig. 5A). We then analyzed by ChIPs NF-Y, Ash2L and H3K4 methylations on CCAAT promoters, in RS4-11 and REH. The levels of H3K4me3 were lower in RS4-11 compared to REH on most, but not all promoters (Fig. 5B). Higher levels of H3K79me2 were found, specifically in the promoters with low levels H3K4me3, which is consistent with previous results with MLL-AF10 fusions [26]–[28]. The H3K4me1 and H3K4me2 levels were extremely low in RS4-11, with the exception of CKS2 and Cyclin B1 promoters. Importantly, the recovery of Ash2L were minimal from all promoters, while NF-Y binding was comparable in the two cell lines, including in promoters with residual levels of H3K4 methylations. We take this as a further indication that H3K4 methylations are not strictly required for NF-Y association. In addition, it is clear that the presence of the oncogenic fusion protein affects the levels of H3K4 mono-, di- and tri-methylation.

## Discussion

### NF-Y and H3K4 methylations

A relevant question in chromatin studies is what determines the location of histone marks on genomes. The binding of TFs and cofactors to promoters are hallmarks of expression, by signalling to the Pol II machinery the appropriate positional coordinates. In general, it seems plausible that TFs are instrumental in determining the positions of specific histone PTMs. However, it is becoming increasingly clear that certain TFs do require a particular set of histone TFs to access regulatory regions: MYC binding, for example, happens only in sites in which H3K4 and H3K79 methylations are abundant [25]. Hence, there must be a hierarchy in TF in establishing a certain chromatin environment, with some TFs being capable to recruit histone modifying enzymes for the benefit of additional TFs. There is a strong correlation in genomic locations of NF-Y and H3K4me3, and removal of NF-Y reduced the local, but not the global levels of H3K4me3 [19]-[24]. Unlike other TFs, the presence of high levels of H3K4me3 is not strictly required for NF-Y recruitment: indeed, NF-Y is involved in the recruitment of Ash2L, indicating that it acts upstream of H3K4me3, and placing it at the heart of the local di- to tri-methylation transition. Furthermore, the results in MLL cells, in which most promoters we analyzed have residual levels of any H3K4 methylation, yet robust NF-Y association, indicate that none of the methylations of H3K4 is strictly required for binding of the trimer to DNA. The fact that H3K4 methylations are downstream of H2B monoubiquitination ([2] and References therein] renders the H2B-like structure of NF-YB particularly relevant, since a parallel signalling can be envisaged.

The presence of the CCAAT box at the top of the list of the Ash2L regulated genes, together with few other TFBS with similar scores, is in line with the data. However, the relatively low statistical enrichments in TFBS analysis among Ash2L-regulated promoters indicate that the preference is not absolute, and many other TFs can recruit Ash2L, as indicated by genetic screenings for interactors of AP2, Mef2c, PAX7 and Tbx1 [13]–[16]. We confirm that removal of Ash2L leads to a drastic decrease in the global levels of H3K4me3, while H3K4me2 and H3K4me1 are not substantially changed. Whether Ash2L is the only subunit of the complex to direct the MLL activity toward trimethylation is currently unclear [9]. As Ash2L is believed to be present in complexes containing different MLLs, we were surprised by the relatively low number of genes affected by Ash2L interference. There are technical explanations for this, such as the incomplete elimination of the protein and the fact that hybridization-derived profiling data are less sensitive to variation with respect to qRT-PCR, suggesting that many additional genes were indeed missed. In addition, the presence of partially redundant activities similar to Ash2L, such as Ash1 [29], should be considered.

### Reciprocal regulation of H3K4me3 and H3K79me2

MLL is a complex and genetically heterogeneous disease in which MLL1 fusion proteins alter gene expression; this is, in part, due to the partners, some of which -AF4, AF9 and ENL- have transcription activation domains required for transformation [10], [27]. In cells bearing AF10 fusions, reduced H3K4me3 was reported to be “compensated” by high H3K79me2 levels. AF10 was shown to bind to hDOT1L, through a domain required for transformation [28]. In accordance with these data, in our analysis of leukemic cells with an MLL1-AF4 fusion, the H3K4me3 decrease is also “compensated” by an increase in H3K79me2. However, in HCT116 cells, which carry a wt MLL1 configuration, the removal of Ash2L alone, by decreasing H3K4me3, is sufficient to increase H3K79me2, and there is an inverse correlation between these two marks on each of the promoters we analyzed. One could therefore hypothesize that it is the absence of Ash2L on promoters, rather than, or in addition to an AF4 or AF10-mediated recruitment of hDOT1L, which leads to high H3K79me2 levels. This finding could have consequences in the temporal deposition of the two marks, which lie downstream of H2BUb, suggesting that H3K79me2 acts upstream of H3K4me3. This matter is further complicated by the fact that mono- and di-methylations are also dramatically affected in MLL cells, which was somewhat expected, based on the assumption that the SET domain is absent from the MLL fusions, but never tested. Importantly, in the absence of the N-terminal end of MLL1, Ash2L is not recruited onto promoters. These data indicate that the oncogenic potential might be influenced by the variation of composition of the MLL complex as a whole, as well as by the presence of the fusion partner.

### A link between the MLL complex and NF-Y in cellular transformation?

In 23 acute lymphoblastic leukemias with MLL translocations, two signatures correlating with prognosis were found [30]. Top rank genes were HspCBF, an NF-Y coactivator [31] in the poor prognosis group, and CDP, a negative regulator of CCAAT activity [32], in the cohort with good prognosis. Importantly, genes with two CCAAT boxes, predicted to be down-regulated by CDP, were found underexpressed in the latter group. (ii) The CCAAT box was repeatedly reported, along with E2F sites, in genes specifically overexpressed in tumors [33]–[35]. Notably, *de novo* motifs discovery in leukemias pointed at three sites: the expected E2F-NF-Y *duo* and p53 [36]. (iii) Analysis of gene expression profiles of MLL-AF9 leukemias identified a haematopoietic stem cell -HSCs- signature that confers self-renewal properties [10], with some targets, such as HOXA members, important for tumor growth. Interestingly, NF-Y was shown to be a potent HSC self-renewal regulator, by activating HOX4 paralogues [37]. Notably, the short form of NF-YA appears to be crucial [38], [39]. Thus, MLL fusions and NF-Y could work together in reactivation of a self-renewal program. The absence of significant levels of H3K4 methylations is coupled to high levels of H3K79me2, a mark also dependent from the presence of NF-Y [24]: we can imagine that NF-Y is also involved in the recruitment of the hDOT1 complex, even without the presence of the AF4/AF10 fusion partner. NF-Y-mediated recruitment of MLL complexes with an altered composition on growth-promoting genes could impair profoundly regulation, *via* alteration of the local histone PTMs. Further biochemical and *in vivo* ChIP work is required to shed light on this hypothesis.

## Materials and Methods

### Cell cultures and transfections

HCT116 cells were cultured in DMEM supplemented with 10% FCS, 1% penicillin and streptomycin, and L-glutamine. All transfections were carried out using Lipofectamine 2000 (Invitrogen, USA). The Ash2L siRNA was purchased by (Dharmacon, USA). Scrambled siRNA was used as a negative control (Ambion, USA). NF-YA and NF-YB shRNA vectors were purchased from Sigma; the control was a similar vector expressing shRNA against GFP.

### RT-PCR analysis, Nuclear and Acid extracts preparation and Western blot analysis

Total RNAs were extracted using an RNA-Easy kit (Qiagen, D). 1µg of each RNA was retrotranscribed (Promega, USA). Normalization of the cDNAs were performed with GAPDH control. The RT-PCR primers used are listed in Figure S3.

Nuclear extracts were prepared according to standard procedures (20). Acid extracts were prepared collecting cells in 5–10 volumes of Lysis Buffer H (10 mM Hepes pH 7.9, 150 mM MgCl_2_, 10 mM KCl, 0.5 mM DTT, 1.5 mM PMSF); Perchloric acid 0.2 M was added and cells were kept on ice for 30 minutes, followed by centrifugation for 10 minutes at 11000 g at 4°C; the supernatants were stocked at −80°C. 15 µg of nuclear and acid extracts were used in 12% SDS-PAGE. Proteins were transferred to nitrocellulose membranes and immunoblotted using the antibodies of interest. The protein-antibody complexes were detected using horseradish peroxidase-conjugated secondary antibodies (GE Healthcare, UK) and the chemioluminescence system (Genespin, I).

### Chromatin Immunoprecipitation

ChIP assays were performed as previously described (Donati et al, 2007). Immunoprecipitations were performed with ProtG-Sepharose (KPL, USA) and 3µg of the following antibodies: NF-YB (Genespin, I); H3 (Abcam 1791); H4K4me3 (Abcam 8580, Active Motif 39159); H4K4me2 (Abcam 7766); H4K4me1 (Abcam 8895); H3K79me2 (Abcam 3594), Ash2L (Active Motif 39099). The MLL antibody was a kind gift of E. Canaani (Weizman I., Il). The ChIP-PCR primers are listed in Figure S3. Quantitative Real Time PCR was performed using SYBR green IQ reagent (Biorad, USA) in the iCycler IQ. The relative sample enrichment was calculated with the following formula: 2 ^ΔCtx^ -2 ^ΔCtb^, where ΔCt x  =  Ct input-Ct sample and ΔCt b  =  Ct input-Ct control Ab.

## Supporting Information

Figure S1 List of Ash2L-regulated genes in HCT116 cells. Genes whose expression is altered upon Ash2L knock down.
(DOC)

Figure S2 Transcription Factor Binding Site (TFBS) analysis of the promoters (-500/+100 from the Transcriptional Start Site) of the Ash2L-regulated genes derived from the profiling experiments.
(DOC)

Figure S3 List of primers used in q-RT-PCR and ChIPs.
(PDF)
