# Supplementary material for: NF-Y Recruits Ash2L to Impart H3K4 Trimethylation on CCAAT Promoters
Source: PLoS One. 2011 Mar 21;6(3):e17220. doi: 10.1371/journal.pone.0017220 (PMC3061855; doi:10.1371/journal.pone.0017220)
Supplement: Figure S1 — List of Ash2L-regulated genes in HCT116 cells. Genes whose expression is altered upon Ash2L knock down. (DOC) [file pone.0017220.s001.doc]

Figure S1

A. Genes downregulated after ASH2L silencing

| SYMBOL | ILMN_GENE | CHROMOSOME | DEFINITION |
| --- | --- | --- | --- |
| ABCA5 | ILMN_3630 | 17 | ATP-binding cassette, sub-family A (ABC1), member 5 (ABCA5). |
| ABHD2 | ILMN_24898 | 15 | abhydrolase domain containing 2 (ABHD2). |
| ACAD8 | ILMN_19744 | 11 | acyl-Coenzyme A dehydrogenase family, member 8 (ACAD8). |
| ACSBG2 | ILMN_138348 | 19 | acyl-CoA synthetase bubblegum family member 2 (ACSBG2). |
| ACSL4 | ILMN_177778 | X | acyl-CoA synthetase long-chain family member 4 (ACSL4). |
| ACSS2 | ILMN_9910 | 20 | acyl-CoA synthetase short-chain family member 2 (ACSS2). |
| ACTA2 | ILMN_6588 | 12 | actin, alpha 2, smooth muscle, aorta (ACTA2). |
| ADAM19 | ILMN_12727 | 5 | ADAM metallopeptidase domain 19 (meltrin beta) (ADAM19). |
| ADAMTS14 | ILMN_176528 | 10 | ADAM metallopeptidase with thrombospondin type 1 motif, 14 (ADAMTS14). |
| ADK | ILMN_29192 | 10 | adenosine kinase (ADK), transcript variant ADK-long. |
| ADPRHL1 | ILMN_2061 | 13 | ADP-ribosylhydrolase like 1 (ADPRHL1). |
| AFAP1L2 | ILMN_23732 | 10 | actin filament associated protein 1-like 2 (AFAP1L2). |
| AKAP11 | ILMN_13368 | 13 | A kinase (PRKA) anchor protein 11 (AKAP11). |
| AKR1B10 | ILMN_6435 | 7 | aldo-keto reductase family 1, member B10 (aldose reductase) (AKR1B10). |
| ALAS1 | ILMN_20926 | 3 | aminolevulinate, delta-, synthase 1 (ALAS1). |
| ALDH1A2 | ILMN_17630 | 15 | aldehyde dehydrogenase 1 family, member A2 (ALDH1A2). |
| ALS2CR14 | ILMN_947 | 2 | amyotrophic lateral sclerosis 2 (juvenile). |
| ALS2CR2 | ILMN_24336 | 19 | amyotrophic lateral sclerosis 2 (juvenile). |
| AMPD3 | ILMN_13634 | 11 | adenosine monophosphate deaminase (isoform E) (AMPD3). |
| ANGPTL4 | ILMN_31891 | 7 | angiopoietin-like 4 (ANGPTL4), transcript variant 3. |
| ANKRD22 | ILMN_7804 | 1 | ankyrin repeat domain 22 (ANKRD22). |
| ANKRD23 | ILMN_32220 | 2 | ankyrin repeat domain 23 (ANKRD23). |
| ANKRD37 | ILMN_2423 |  | ankyrin repeat domain 37 (ANKRD37). |
| ANKRD5 | ILMN_9924 | 20 | ankyrin repeat domain 5 (ANKRD5). |
| AP3M1 | ILMN_5879 | 10 | adaptor-related protein complex 3, mu 1 subunit (AP3M1). |
| APAF1 | ILMN_886 | 12 | apoptotic peptidase activating factor 1 (APAF1). |
| APOBEC3B | ILMN_5305 | 22 | apolipoprotein B editing enzyme, catalytic polypeptide-like 3B (APOBEC3B). |
| APOL2 | ILMN_19374 | 22 | apolipoprotein L, 2 (APOL2), transcript variant alpha. |
| ARHGDIB | ILMN_9074 | 11 | Rho GDP dissociation inhibitor (GDI) beta (ARHGDIB). |
| ARHGEF16 | ILMN_3096 | 1 | Rho guanine exchange factor (GEF) 16 (ARHGEF16). |
| ARL4C | ILMN_15416 | 2 | ADP-ribosylation factor-like 4C (ARL4C). |
| ARMCX3 | ILMN_15168 | X | armadillo repeat containing, X-linked 3 (ARMCX3). |
| ASH2L | ILMN_14109 | 2 | ash2 (absent, small, or homeotic)-like (Drosophila) (ASH2L). |
| ASPHD1 | ILMN_11756 | 16 | aspartate beta-hydroxylase domain containing 1 (ASPHD1). |
| ATL3 | ILMN_20827 | 2 | atlastin 3 (ATL3). |
| ATM | ILMN_16722 | 11 | ataxia telangiectasia mutated (ATM). |
| ATP2A3 | ILMN_26846 | 17 | ATPase, Ca++ transporting, ubiquitous (ATP2A3). |
| BAI1 | ILMN_25426 | 8 | brain-specific angiogenesis inhibitor 1 (BAI1). |
| BAX | ILMN_11763 | 19 | BCL2-associated X protein (BAX), transcript variant sigma. |
| BBS9 | ILMN_165104 | 7 | Bardet-Biedl syndrome 9 (BBS9). |
| BCL2L11 | ILMN_16692 | 2 | BCL2-like 11 (apoptosis facilitator) (BCL2L11), transcript variant 9. |
| BCL6 | ILMN_540 | 3 | B-cell CLL/lymphoma 6 (zinc finger protein 51) (BCL6). |
| BIRC7 | ILMN_21032 | 20 | baculoviral IAP repeat-containing 7 (livin) (BIRC7). |
| BLVRB | ILMN_11803 | 10 | biliverdin reductase B (flavin reductase (NADPH)) (BLVRB). |
| BTBD3 | ILMN_22115 | 20 | BTB (POZ) domain containing 3 (BTBD3). |
| BTRC | ILMN_25556 | 10 | beta-transducin repeat containing (BTRC). |
| C10ORF10 | ILMN_8623 | 8 | chromosome 10 open reading frame 10 (C10orf10). |
| C10orf11 | ILMN_17394 | 10 | chromosome 10 open reading frame 11 (C10orf11). |
| C10orf12 | ILMN_15899 | 10 | chromosome 10 open reading frame 12 (C10orf12). |
| C10orf4 | ILMN_549 | 10 | chromosome 10 open reading frame 4 (C10orf4). |
| C10orf61 | ILMN_7866 | 10 | chromosome 10 open reading frame 61 (C10orf61). |
| C14orf143 | ILMN_13424 | 14 | chromosome 14 open reading frame 143 (C14orf143). |
| C14orf151 | ILMN_6769 | 14 | chromosome 14 open reading frame 151 (C14orf151). |
| C14ORF151 | ILMN_6769 | 3 | chromosome 14 open reading frame 151 (C14orf151). |
| C14orf21 | ILMN_9819 | 14 | chromosome 14 open reading frame 21 (C14orf21). |
| C14orf45 | ILMN_24020 | 14 | chromosome 14 open reading frame 45 (C14orf45). |
| C15orf38 | ILMN_18724 | 15 | chromosome 15 open reading frame 38 (C15orf38). |
| C15ORF41 | ILMN_18248 | 14 | chromosome 15 open reading frame 41 (C15orf41). |
| C16orf35 | ILMN_42546 | 16 | chromosome 16 open reading frame 35 (C16orf35). |
| C19orf45 | ILMN_16641 | 19 | chromosome 19 open reading frame 45 (C19orf45). |
| C1ORF66 | ILMN_20031 | 17 | chromosome 1 open reading frame 66 (C1orf66). |
| C1orf84 | ILMN_10202 | 1 | chromosome 1 open reading frame 84 (C1orf84). |
| C20orf107 | ILMN_1548 | 20 | chromosome 20 open reading frame 107 (C20orf107). |
| C20orf195 | ILMN_7222 | 20 | chromosome 20 open reading frame 195 (C20orf195). |
| C2ORF15 | ILMN_16952 | 11 | chromosome 2 open reading frame 15 (C2orf15). |
| C3orf18 | ILMN_9603 | 3 | chromosome 3 open reading frame 18 (C3orf18). |
| C3orf63 | ILMN_25103 | 3 | chromosome 3 open reading frame 63 (C3orf63). |
| C4orf36 | ILMN_5377 | 4 | chromosome 4 open reading frame 36 (C4orf36). |
| C4orf38 | ILMN_24921 | 4 | chromosome 4 open reading frame 38 (C4orf38). |
| C5orf34 | ILMN_179972 | 5 | chromosome 5 open reading frame 34 (C5orf34). |
| C5orf39 | ILMN_17443 | 5 | chromosome 5 open reading frame 39 (C5orf39). |
| C6ORF166 | ILMN_1311 | 8 | chromosome 6 open reading frame 166 (C6orf166). |
| C6ORF57 | ILMN_23605 | 10 | chromosome 6 open reading frame 57 (C6orf57). |
| C8orf13 | ILMN_27702 | 8 | chromosome 8 open reading frame 13 (C8orf13). |
| C8ORF40 | ILMN_20841 | 3 | chromosome 8 open reading frame 40 (C8orf40). |
| C9ORF119 | ILMN_34116 | 1 | chromosome 9 open reading frame 119 (C9orf119). |
| C9orf165 | ILMN_13417 | 9 | chromosome 9 open reading frame 165 (C9orf165). |
| C9orf66 | ILMN_3927 | 9 | chromosome 9 open reading frame 66 (C9orf66). |
| C9orf84 | ILMN_17236 | 9 | chromosome 9 open reading frame 84 (C9orf84). |
| C9ORF9 | ILMN_12570 |  | chromosome 9 open reading frame 9 (C9orf9). |
| CALB2 | ILMN_11415 | 10 | calbindin 2, 29kDa (calretinin) (CALB2), transcript variant CALB2c. |
| CALN1 | ILMN_27637 | 7 | calneuron 1 (CALN1). |
| CATSPER1 | ILMN_5805 | 11 | cation channel, sperm associated 1 (CATSPER1). |
| CCDC110 | ILMN_15423 | 4 | coiled-coil domain containing 110 (CCDC110). |
| CCDC117 | ILMN_170970 | 22 | coiled-coil domain containing 117 (CCDC117). |
| CCDC34 | ILMN_2645 | 11 | coiled-coil domain containing 34 (CCDC34). |
| CCDC89 | ILMN_4285 | 11 | coiled-coil domain containing 89 (CCDC89). |
| CCNB2 | ILMN_15254 | 3 | cyclin B2 (CCNB2). |
| CCNT2 | ILMN_11186 | 2 | cyclin T2 (CCNT2), transcript variant a. |
| CD300LG | ILMN_20387 | 17 | CD300 molecule-like family member g (CD300LG). |
| CD68 | ILMN_5188 | 9 | CD68 molecule (CD68), transcript variant 1. |
| CD70 | ILMN_19983 | 19 | CD70 molecule (CD70). |
| CDA | ILMN_137423 | 16 | cytidine deaminase (CDA). |
| CDC25B | ILMN_137183 | 20 | cell division cycle 25B (CDC25B), transcript variant 4. |
| CDH24 | ILMN_791 | 14 | cadherin-like 24 (CDH24). |
| CDK6 | ILMN_3062 | 19 | cyclin-dependent kinase 6 (CDK6). |
| CDKN2B | ILMN_183997 | 9 | cyclin-dependent kinase inhibitor 2B (p15, inhibits CDK4) (CDKN2B). |
| CDS2 | ILMN_18323 | 19 | CDP-diacylglycerol synthase (phosphatidate cytidylyltransferase) 2 (CDS2). |
| CENPH | ILMN_18041 | 5 | centromere protein H (CENPH). |
| CEP192 | ILMN_7783 | 18 | centrosomal protein 192kDa (CEP192). |
| CEP57 | ILMN_27141 | 11 | centrosomal protein 57kDa (CEP57). |
| CGI-96 | ILMN_12816 | 22 | CGI-96 protein (CGI-96). |
| CHEK2 | ILMN_4865 | 22 | CHK2 checkpoint homolog (S. pombe) (CHEK2). |
| CITED4 | ILMN_15271 | 11 | Cbp/p300-interacting transactivator, with Glu/Asp-rich carboxy-terminal domain. |
| CLEC2D | ILMN_15059 | 12 | C-type lectin domain family 2, member D (CLEC2D). |
| CLPB | ILMN_2118 | 11 | ClpB caseinolytic peptidase B homolog (E. coli) (CLPB). |
| CMTM1 | ILMN_8242 | 16 | CKLF-like MARVEL transmembrane domain containing 1 (CMTM1). |
| CNOT7 | ILMN_137418 | 8 | CCR4-NOT transcription complex, subunit 7 (CNOT7). |
| CNTNAP5 | ILMN_12389 | 2 | contactin associated protein-like 5 (CNTNAP5). |
| COL17A1 | ILMN_5067 | 10 | collagen, type XVII, alpha 1 (COL17A1). |
| COX17 | ILMN_19252 | 9 | COX17 cytochrome c oxidase assembly homolog (S, cerevisiae) (COX17). |
| CPA4 | ILMN_21403 | 1 | carboxypeptidase A4 (CPA4). |
| CPEB2 | ILMN_3402 | 4 | cytoplasmic polyadenylation element binding protein 2 (CPEB2). |
| CPZ | ILMN_14396 | 4 | carboxypeptidase Z (CPZ). |
| CSNK1E | ILMN_24481 | 22 | casein kinase 1, epsilon (CSNK1E). |
| CSNK1G2 | ILMN_17274 | 14 | casein kinase 1, gamma 2 (CSNK1G2). |
| CYB561 | ILMN_9883 | 17 | cytochrome b-561 (CYB561). |
| CYB5D2 | ILMN_26268 | 11 | cytochrome b5 domain containing 2 (CYB5D2). |
| CYBRD1 | ILMN_4649 | X | cytochrome b reductase 1 (CYBRD1). |
| CYP4F2 | ILMN_18195 | 19 | cytochrome P450, family 4, subfamily F, polypeptide 2 (CYP4F2). |
| DAG1 | ILMN_16432 | 3 | dystroglycan 1 (dystrophin-associated glycoprotein 1) (DAG1). |
| DBP | ILMN_1724 | 19 | D site of albumin promoter (albumin D-box) binding protein (DBP). |
| DCP2 | ILMN_4971 | 17 | DCP2 decapping enzyme homolog (S, cerevisiae) (DCP2). |
| DCTN3 | ILMN_2126 | 9 | dynactin 3 (p22) (DCTN3). |
| DDI2 | ILMN_27420 | 1 | DDI1, DNA-damage inducible 1, homolog 2 (S. cerevisiae) (DDI2). |
| DDIT4 | ILMN_13176 | 2 | DNA-damage-inducible transcript 4 (DDIT4). |
| DDX12 | ILMN_34429 | 12 | PREDICTED: DEAD/H (Asp-Glu-Ala-Asp/His) box polypeptide 12. |
| DDX60 | ILMN_17673 | 4 | DEAD (Asp-Glu-Ala-Asp) box polypeptide 60 (DDX60). |
| DEFB131 | ILMN_166014 | 4 | defensin, beta 131 (DEFB131). |
| DHRS3 | ILMN_12432 | X | dehydrogenase/reductase (SDR family) member 3 (DHRS3). |
| DICER1 | ILMN_24464 | 14 | Dicer1, Dcr-1 homolog (Drosophila) (DICER1). |
| DKFZp434K191 | ILMN_28495 | 22 | hypothetical protein DKFZp434K191 (DKFZp434K191). |
| DKFZp686O24166 | ILMN_19977 | 11 | hypothetical protein DKFZp686O24166 (DKFZp686O24166). |
| DKK1 | ILMN_22862 | 15 | dickkopf homolog 1 (Xenopus laevis) (DKK1). |
| DNAJC16 | ILMN_139021 | 1 | DnaJ (Hsp40) homolog, subfamily C, member 16 (DNAJC16). |
| DNASE1 | ILMN_14833 | 16 | deoxyribonuclease I (DNASE1). |
| DNASE2B | ILMN_27910 | 1 | deoxyribonuclease II beta (DNASE2B). |
| DPH1 | ILMN_21347 | 17 | DPH1 homolog (S. cerevisiae) (DPH1). |
| DSCAM | ILMN_1163 | 21 | Down syndrome cell adhesion molecule (DSCAM). |
| DTX2 | ILMN_21612 | 7 | deltex homolog 2 (Drosophila) (DTX2). |
| DUSP2 | ILMN_21154 | 2 | dual specificity phosphatase 2 (DUSP2). |
| ECE2 | ILMN_175313 | 3 | endothelin converting enzyme 2 (ECE2). |
| EFEMP2 | ILMN_137851 | 11 | EGF-containing fibulin-like extracellular matrix protein 2 (EFEMP2). |
| EGR2 | ILMN_10721 | 10 | early growth response 2 (Krox-20 homolog, Drosophila) (EGR2). |
| EIF4EBP2 | ILMN_16446 | 6 | eukaryotic translation initiation factor 4E binding protein 2 (EIF4EBP2). |
| EIF5A2 | ILMN_4591 | 3 | eukaryotic translation initiation factor 5A2 (EIF5A2). |
| EPB41 | ILMN_15301 | 1 | erythrocyte membrane protein band 4.1 (elliptocytosis 1, RH-linked) (EPB41). |
| EPM2A | ILMN_16230 | 6 | epilepsy, progressive myoclonus type 2A, Lafora disease (laforin) (EPM2A). |
| EPS8L1 | ILMN_15325 | 19 | EPS8-like 1 (EPS8L1). |
| ERC1 | ILMN_32989 | 12 | ELKS/RAB6-interacting/CAST family member 1 (ERC1), transcript variant gamma. |
| ERGIC1 | ILMN_7272 | 5 | endoplasmic reticulum-golgi intermediate compartment (ERGIC) 1 (ERGIC1). |
| FAM100B | ILMN_22874 | 6 | family with sequence similarity 100, member B (FAM100B). |
| FAM127A | ILMN_27078 | 3 | family with sequence similarity 127, member A (FAM127A). |
| FAM129B | ILMN_8005 | 9 | family with sequence similarity 129, member B (FAM129B). |
| FAM53B | ILMN_165220 | 10 | family with sequence similarity 53, member B (FAM53B). |
| FAM73A | ILMN_4385 | 1 | family with sequence similarity 73, member A (FAM73A). |
| FBXO16 | ILMN_1262 | 3 | F-box protein 16 (FBXO16). |
| FBXO17 | ILMN_20838 | 19 | F-box protein 17 (FBXO17). |
| FBXO24 | ILMN_3577 | 7 | F-box protein 24 (FBXO24). |
| FBXO27 | ILMN_14974 | 19 | F-box protein 27 (FBXO27). |
| FBXO27 | ILMN_14974 | 7 | F-box protein 27 (FBXO27). |
| FBXW8 | ILMN_4038 | 12 | F-box and WD repeat domain containing 8 (FBXW8). |
| FGFR3 | ILMN_22960 | 4 | fibroblast growth factor receptor 3. |
| FHIT | ILMN_15082 | 3 | fragile histidine triad gene (FHIT). |
| FLJ10374 | ILMN_3627 | 19 | hypothetical protein FLJ10374 (FLJ10374). |
| FLJ30058 | ILMN_29759 | X | hypothetical protein FLJ30058 (FLJ30058). |
| FLJ35767 | ILMN_28580 | 6 | FLJ35767 protein (FLJ35767). |
| FLJ38717 | ILMN_13488 | 6 | FLJ38717 protein (FLJ38717). |
| FLJ39061 | ILMN_172404 |  | hypothetical protein FLJ39061 (FLJ39061). |
| FRYL | ILMN_138098 | 4 | FRY-like (FRYL). |
| FRZB | ILMN_29091 | 2 | frizzled-related protein (FRZB). |
| FUSIP1 | ILMN_30145 | 1 | FUS interacting protein (serine/arginine-rich) 1 (FUSIP1). |
| GALE | ILMN_24444 | 1 | UDP-galactose-4-epimerase (GALE). |
| GCUD2 | ILMN_19354 | 1|NT_113874.1 | gastric cancer up-regulated-2 (GCUD2). |
| GLB1 | ILMN_23626 | 3 | galactosidase, beta 1 (GLB1), transcript variant 179423. |
| GLUL | ILMN_25881 | 1 | glutamate-ammonia ligase (glutamine synthetase) (GLUL), transcript variant 3. |
| GNAT1 | ILMN_21210 | 3 | guanine nucleotide binding protein (G protein). |
| GOLGA6B | ILMN_6462 | 15 | golgi autoantigen, golgin subfamily a, 6B (GOLGA6B). |
| GPNMB | ILMN_25675 | 17 | glycoprotein (transmembrane) nmb (GPNMB), transcript variant 2. |
| GPR161 | ILMN_22837 | 1 | G protein-coupled receptor 161 (GPR161). |
| GPX1 | ILMN_10376 | 3 | glutathione peroxidase 1 (GPX1). |
| GRB10 | ILMN_2830 | 7 | growth factor receptor-bound protein 10 (GRB10). |
| GRINL1A | ILMN_20762 | 15 | glutamate receptor, ionotropic, N-methyl D-aspartate-like 1A (GRINL1A). |
| H1FX | ILMN_26614 |  | H1 histone family, member X (H1FX). |
| H3F3B | ILMN_26885 |  | H3 histone, family 3B (H3,3B) (H3F3B). |
| HACL1 | ILMN_24469 | 9 | 2-hydroxyacyl-CoA lyase 1 (HACL1). |
| HDAC7A | ILMN_29995 | 12 | histone deacetylase 7A (HDAC7A), transcript variant 3. |
| HESX1 | ILMN_23312 | 3 | HESX homeobox 1 (HESX1). |
| HESX1 | ILMN_23312 | 11 | HESX homeobox 1 (HESX1). |
| HEXIM2 | ILMN_12244 |  | hexamthylene bis-acetamide inducible 2 (HEXIM2). |
| HIST1H2AM | ILMN_26622 | 6 | histone cluster 1, H2am (HIST1H2AM). |
| HIST1H2BM | ILMN_27454 | 6 | histone cluster 1, H2bm (HIST1H2BM). |
| HIST2H2BE | ILMN_28293 | 1 | histone cluster 2, H2be (HIST2H2BE). |
| HLA-DMB | ILMN_2252 | 6 | major histocompatibility complex, class II, DM beta (HLA-DMB). |
| HMGCL | ILMN_18301 | 3 | 3-hydroxymethyl-3-methylglutaryl-Coenzyme A lyase. |
| HNRNPA3P1 | ILMN_5777 | 10 | heterogeneous nuclear ribonucleoprotein A3 pseudogene 1 (HNRNPA3P1) |
| HOXA4 | ILMN_138508 | 7 | homeobox A4 (HOXA4). |
| HOXB1 | ILMN_138553 | 17 | homeobox B1 (HOXB1). |
| HOXC6 | ILMN_172478 | 12 | homeobox C6 (HOXC6). |
| HS,12876 | ILMN_71296 | 10 | 602659965F1 NCI_CGAP_Skn3 cDNA clone IMAGE:4802969 5, sequence |
| HS,279842 | ILMN_84472 | 17 | HSPC157 protein, (cDNA clone IMAGE:6672800), partial cds |
| HS,326560 | ILMN_86232 | 7 | PREDICTED: LOC440151 (LOC440151), |
| HS,386232 | ILMN_89152 | 9 | RST21348 Athersys RAGE Library cDNA, sequence |
| HS,568690 | ILMN_120871 | 12 | PM0-ST0264-161199-001-b06 ST0264 cDNA, sequence |
| HS,568741 | ILMN_120922 | 7 | TC125227 Human breast cancer tissue. |
| HS,569162 | ILMN_121343 | 6 | AV681673 GKB cDNA clone GKBABD06 5, sequence |
| HS,574590 | ILMN_126771 | 19 | DA728582 NT2RM2 cDNA clone NT2RM2002174 5, sequence |
| HS.120300 | ILMN_75629 |  | HESC4_33_E11.g1_A037 NIH_MGC_262 cDNA clone IMAGE:7474271 5. |
| HS.123214 | ILMN_75922 |  | hd27b07.y1 Human Retina cDNA. |
| HS.125056 | ILMN_76084 |  | cDNA FLJ36663 fis, clone UTERU2002826 |
| HS.144479 | ILMN_78077 |  | AGENCOURT_13979145 NIH_MGC_179 cDNA clone IMAGE:30367627 5. |
| HS.145444 | ILMN_78206 |  | cDNA FLJ11494 fis, clone HEMBA1001942 |
| HS.167721 | ILMN_80111 |  | cDNA FLJ37425 fis, clone BRAWH2001530 |
| HS.168162 | ILMN_80119 |  | AGENCOURT_13779116 NIH_MGC_184 cDNA clone IMAGE:30349586 5. |
| HS.23459 | ILMN_71835 |  | PREDICTED: hypothetical LOC388727 (LOC388727), |
| HS.245405 | ILMN_83114 |  | UI-H-CO0-ara-d-07-0-UI.s1 NCI_CGAP_Sub9 cDNA clone IMAGE:3105827 3. |
| HS.412918 | ILMN_90317 |  | cDNA FLJ32550 fis, clone SPLEN1000056 |
| HS.445843 | ILMN_93075 |  | 602617110F1 NIH_MGC_79 cDNA clone IMAGE:4730811 5. |
| HS.518527 | ILMN_99459 |  | PREDICTED: hypothetical LOC389189 (LOC389189), |
| HS.527515 | ILMN_100905 |  | AGENCOURT_13631433 NIH_MGC_184 cDNA clone IMAGE:30327753 5. |
| HS.539765 | ILMN_104729 |  | 601823962F1 NIH_MGC_79 cDNA clone IMAGE:4043678 5. |
| HS.548213 | ILMN_109801 |  | tx54c03.x1 NCI_CGAP_Lu24 cDNA clone IMAGE:2273380 3. |
| HS.561526 | ILMN_114927 |  | full-length cDNA clone CS0DN003YC08 of Adult brain of (human) |
| HS.569175 | ILMN_121356 |  | DA697821 NT2NE2 cDNA clone NT2NE2019092 5, sequence |
| HS.569953 | ILMN_122134 |  | MR3-FN0206-070201-014-b05 FN0206 cDNA, sequence |
| HS.571028 | ILMN_123209 |  | 17000531973183 GRN_ES cDNA 5, sequence |
| HS.571741 | ILMN_123922 |  | ny62g05.s1 NCI_CGAP_GCB1 cDNA clone IMAGE:1282904 3, sequence |
| HS.572064 | ILMN_124245 |  | UI-E-EJ1-ajh-l-06-0-UI.r1 UI-E-EJ1 cDNA clone UI-E-EJ1-ajh-l-06-0-UI 5. |
| HS.575085 | ILMN_127266 |  | DB336481 TESTI2 cDNA clone TESTI2007279 3, sequence |
| HS.98330 | ILMN_74482 |  | PREDICTED: hypothetical LOC388227 (LOC388227), |
| ID1 | ILMN_28002 | 5 | inhibitor of DNA binding 1, dominant negative helix-loop-helix protein (ID1). |
| IGSF8 | ILMN_5527 | 1 | immunoglobulin superfamily, member 8 (IGSF8). |
| IL15RA | ILMN_4552 | 10 | interleukin 15 receptor, alpha (IL15RA). |
| IL18BP | ILMN_10368 | 11 | interleukin 18 binding protein (IL18BP), transcript variant D. |
| IL6R | ILMN_22419 | 1 | interleukin 6 receptor (IL6R). |
| INPP5A | ILMN_137853 |  | PREDICTED: inositol polyphosphate-5-phosphatase, 40kDa (INPP5A). |
| KAZALD1 | ILMN_28149 | 10 | Kazal-type serine peptidase inhibitor domain 1 (KAZALD1). |
| KCNH6 | ILMN_171630 | 17 | potassium voltage-gated channel, subfamily H (eag-related), member 6 (KCNH6). |
| KCNJ1 | ILMN_19663 | 11 | potassium inwardly-rectifying channel, subfamily J, member 1 (KCNJ1). |
| KCNN4 | ILMN_11994 |  | potassium intermediate/small conductance calcium-activated channel. |
| KIAA0247 | ILMN_2972 | 14 | KIAA0247 (KIAA0247). |
| KIAA1107 | ILMN_33304 |  | PREDICTED: KIAA1107 (KIAA1107). |
| KIAA1539 | ILMN_29031 | 17 | KIAA1539 (KIAA1539). |
| KLF9 | ILMN_2670 | 6 | Kruppel-like factor 9 (KLF9). |
| KLHDC9 | ILMN_13602 | 1 | kelch domain containing 9 (KLHDC9). |
| KLK8 | ILMN_21619 | 19 | kallikrein-related peptidase 8 (KLK8). |
| KRT15 | ILMN_3189 |  | keratin 15 (KRT15). |
| KRT9 | ILMN_15527 | 17 | keratin 9 (epidermolytic palmoplantar keratoderma) (KRT9). |
| LEMD1 | ILMN_28526 | 19 | LEM domain containing 1 (LEMD1). |
| LEPREL1 | ILMN_11123 | 3 | leprecan-like 1 (LEPREL1). |
| LILRB3 | ILMN_14901 | 19 | leukocyte immunoglobulin-like receptor, subfamily B (with TM and ITIM domains). |
| LIMA1 | ILMN_6603 | 12 | LIM domain and actin binding 1 (LIMA1). |
| LIPH | ILMN_17820 | 3 | lipase, member H (LIPH). |
| LMTK3 | ILMN_35173 | 20 | PREDICTED: lemur tyrosine kinase 3 (LMTK3). |
| LOC144383 | ILMN_41654 |  | PREDICTED: similar to Interferon-induced transmembrane protein 3. |
| LOC144383 | ILMN_41654 | 19 | PREDICTED: similar to Interferon-induced transmembrane protein 3. |
| LOC150383 | ILMN_11584 | 22 | similar to RIKEN cDNA 2210021J22 (LOC150383). |
| LOC286310 | ILMN_34458 | 9 | PREDICTED: lipocalin 1-like 1 (LOC286310), misc RNA. |
| LOC338758 | ILMN_37634 | 12 | PREDICTED: hypothetical protein LOC338758 (LOC338758). |
| LOC388344 | ILMN_34544 | 17 | PREDICTED: similar to ribosomal protein L13, transcript variant 1 (LOC388344). |
| LOC390561 | ILMN_138198 | 15 | PREDICTED: similar to hect domain and RLD 2 (LOC390561). |
| LOC390637 | ILMN_16995 | 15 | similar to RIKEN cDNA D330012F22 gene (LOC390637). |
| LOC400214 | ILMN_42653 |  | PREDICTED: hypothetical gene supported by BX248296 (LOC400214). |
| LOC401677 | ILMN_36492 |  | PREDICTED: similar to eukaryotic translation elongation factor 1 alpha 2 (LOC401677). |
| LOC441294 | ILMN_169689 | 7 | similar to CTAGE6 (LOC441294). |
| LOC442597 | ILMN_42881 |  | PREDICTED: hypothetical LOC442597 (LOC442597). |
| LOC51035 | ILMN_13595 | 15 | SAPK substrate protein 1 (LOC51035). |
| LOC641978 | ILMN_31151 |  | PREDICTED: similar to general transcription factor II I (LOC641978). |
| LOC642035 | ILMN_33714 | 5 | PREDICTED: hypothetical protein LOC642035 (LOC642035). |
| LOC642393 | ILMN_41363 | 1 | PREDICTED: similar to mitochondrial ribosomal protein L20, transcript variant 2. |
| LOC642726 | ILMN_39041 | 4 | PREDICTED: hypothetical protein LOC642725, transcript variant 1 (LOC642726). |
| LOC642969 | ILMN_31159 | 12 | PREDICTED: similar to Phosphoglycerate mutase 1. |
| LOC643272 | ILMN_32441 | 10 | PREDICTED: hypothetical protein LOC643272 (LOC643272). |
| LOC644889 | ILMN_33330 | 11 | PREDICTED: similar to large subunit ribosomal protein L36a (LOC644889). |
| LOC645676 | ILMN_41958 | 1 | PREDICTED: hypothetical protein LOC645676, transcript variant 1 (LOC645676). |
| LOC653604 | ILMN_40991 | 1 | PREDICTED: similar to H3 histone, family 2 isoform 2 (LOC653604). |
| LOC654126 | ILMN_40336 |  | PREDICTED: similar to leucine rich repeat containing 37B. |
| LOC729776 | ILMN_102997 | 9 | PREDICTED: hypothetical protein LOC729776 (LOC729776). |
| LOC730083 | ILMN_32599 | 16 | PREDICTED: similar to exonuclease domain containing 1 (LOC730083). |
| LOC731950 | ILMN_138552 |  | PREDICTED: similar to slit (Drosophila) homolog 2 (LOC731950). |
| LRRC1 | ILMN_13909 | 6 | leucine rich repeat containing 1 (LRRC1). |
| LRRC29 | ILMN_11967 | 16 | leucine rich repeat containing 29 (LRRC29). |
| LRRC59 | ILMN_26734 | 17 | leucine rich repeat containing 59 (LRRC59). |
| LRRFIP2 | ILMN_5592 | 3 | leucine rich repeat (in FLII) interacting protein 2 (LRRFIP2). |
| LSM6 | ILMN_8596 | 4 | LSM6 homolog, U6 small nuclear RNA associated (S. cerevisiae) (LSM6). |
| LYZ | ILMN_4879 | 12 | lysozyme (renal amyloidosis) (LYZ). |
| M6PRBP1 | ILMN_10971 | 4 | mannose-6-phosphate receptor binding protein 1 (M6PRBP1). |
| MACF1 | ILMN_3751 | 1 | microtubule-actin crosslinking factor 1 (MACF1). |
| MADD | ILMN_9428 | 11 | MAP-kinase activating death domain (MADD), transcript variant 5. |
| MAGEH1 | ILMN_8979 | X | melanoma antigen family H, 1 (MAGEH1). |
| MAP2 | ILMN_38764 | 2 | microtubule-associated protein 2 (MAP2). |
| MAP3K3 | ILMN_426 | 17 | mitogen-activated protein kinase kinase kinase 3 (MAP3K3). |
| MAPK14 | ILMN_17267 | 6 | mitogen-activated protein kinase 14 (MAPK14). |
| MAPK7 | ILMN_9862 | 17 | mitogen-activated protein kinase 7 (MAPK7), transcript variant 3. |
| MAX | ILMN_2124 | 14 | MYC associated factor X (MAX), transcript variant 3. |
| ME3 | ILMN_24802 | 11 | malic enzyme 3, NADP(+)-dependent, mitochondrial (ME3). |
| MGAT5 | ILMN_21616 | 2 | mannosyl (alpha-1,6-)-glycoprotein beta-1,6-N-acetyl-glucosaminyltransferase. |
| MGC3207 | ILMN_138056 | 19 | hypothetical protein MGC3207 (MGC3207). |
| MGC59937 | ILMN_13120 | 9 | Similar to RIKEN cDNA 2310002J15 gene (MGC59937). |
| MGC59937 | ILMN_13120 | 19 | Similar to RIKEN cDNA 2310002J15 gene (MGC59937). |
| MGC70863 | ILMN_10471 | 22 | similar to RPL23AP7 protein (MGC70863). |
| MIB2 | ILMN_139150 |  | PREDICTED: mindbomb homolog 2 (Drosophila) (MIB2). |
| MIDN | ILMN_6472 | 14 | midnolin (MIDN). |
| MLYCD | ILMN_2280 | 16 | malonyl-CoA decarboxylase(MLYCD), nuclear gene encoding mitochondrial protein. |
| MMP7 | ILMN_9188 | 11 | matrix metallopeptidase 7 (matrilysin, uterine) (MMP7). |
| MMP9 | ILMN_28136 | 20 | matrix metallopeptidase 9. |
| MRPL40 | ILMN_21771 |  | mitochondrial ribosomal protein L40 (MRPL40). |
| MUC1 | ILMN_162845 | 1 | mucin 1, cell surface associated (MUC1), transcript variant 5. |
| MUC4 | ILMN_164899 | 3 | mucin 4, cell surface associated (MUC4). |
| MYEOV | ILMN_4623 | 8 | myeloma overexpressed gene (in a subset of t(11;14). |
| MZF1 | ILMN_8368 | 19 | myeloid zinc finger 1 (MZF1). |
| NAT5 | ILMN_43222 | 20 | N-acetyltransferase 5 (NAT5), transcript variant 3. |
| NAT6 | ILMN_29898 | 17 | N-acetyltransferase 6 (NAT6). |
| NBPF10 | ILMN_137199 | 1 | PREDICTED: neuroblastoma breakpoint family. |
| NBR2 | ILMN_1966 | 17 | neighbor of BRCA1 gene 2 (NBR2). |
| NDUFS2 | ILMN_9109 | 1 | NADH dehydrogenase (ubiquinone) Fe-S protein 2, 49kDa. |
| NEDD4 | ILMN_27071 | 15 | neural precursor cell expressed, developmentally down-regulated 4 (NEDD4). |
| NEIL1 | ILMN_18060 | 15 | nei endonuclease VIII-like 1 (E. coli) (NEIL1). |
| NEK11 | ILMN_10349 | 3 | NIMA (never in mitosis gene a)- related kinase 11 (NEK11). |
| NEK2 | ILMN_14211 | 19 | NIMA (never in mitosis gene a)-related kinase 2 (NEK2). |
| NOPE | ILMN_17897 | 15 | neighbor of Punc E11 (NOPE). |
| NSMCE2 | ILMN_15101 | 10 | non-SMC element 2, MMS21 homolog (S, cerevisiae) (NSMCE2). |
| NT5E | ILMN_28610 | 10 | 5'-nucleotidase, ecto (CD73) (NT5E). |
| NT5M | ILMN_14877 | 17 | 5',3'-nucleotidase, mitochondrial (NT5M). |
| NUBP2 | ILMN_10701 | 2 | nucleotide binding protein 2 (MinD homolog, E, coli) (NUBP2). |
| OLFML2B | ILMN_572 | 1 | olfactomedin-like 2B (OLFML2B). |
| OR1J1 | ILMN_13947 | 9 | olfactory receptor, family 1, subfamily J, member 1 (OR1J1). |
| OR2A20P | ILMN_13267 | 7 | olfactory receptor, family 2, subfamily A, member 20 pseudogene. |
| OR51B2 | ILMN_20840 |  | olfactory receptor, family 51, subfamily B, member 2 (OR51B2). |
| OR51F1 | ILMN_19096 | 11 | olfactory receptor, family 51, subfamily F, member 1 (OR51F1). |
| OR51I1 | ILMN_10084 | 11 | olfactory receptor, family 51, subfamily I, member 1 (OR51I1). |
| P2RY2 | ILMN_15883 | 11 | purinergic receptor P2Y, G-protein coupled, 2 (P2RY2). |
| PAG1 | ILMN_174074 | 8 | phosphoprotein associated with glycosphingolipid microdomains 1 (PAG1). |
| PARD6G | ILMN_138732 |  | PREDICTED: par-6 partitioning defective 6 homolog gamma (C. elegans). |
| PAX8 | ILMN_6456 | 2 | paired box 8 (PAX8), transcript variant PAX8D. |
| PBRM1 | ILMN_16253 | 3 | polybromo 1 (PBRM1), transcript variant 4. |
| PCOLCE | ILMN_13969 | 1 | procollagen C-endopeptidase enhancer (PCOLCE). |
| PCTK1 | ILMN_11214 | X | PCTAIRE protein kinase 1 (PCTK1). |
| PDE4C | ILMN_29696 | 19 | phosphodiesterase 4C, cAMP-specific. |
| PERP | ILMN_4512 | 16 | PERP, TP53 apoptosis effector (PERP). |
| PGBD4 | ILMN_6577 | 15 | piggyBac transposable element derived 4 (PGBD4). |
| PIGX | ILMN_21837 | 3 | phosphatidylinositol glycan anchor biosynthesis, class X (PIGX). |
| PLA2G6 | ILMN_13517 | 22 | phospholipase A2, group VI (cytosolic, calcium-independent) (PLA2G6). |
| PLEKHH2 | ILMN_28132 | 2 | pleckstrin homology domain containing, family H (with MyTH4 domain). |
| PMEPA1 | ILMN_24935 | 20 | prostate transmembrane protein, androgen induced 1 (PMEPA1). |
| PMP22 | ILMN_9212 | 17 | peripheral myelin protein 22 (PMP22). |
| PODXL | ILMN_24120 | 7 | podocalyxin-like (PODXL). |
| POLD4 | ILMN_14887 | 17 | polymerase (DNA-directed), delta 4 (POLD4). |
| POLR2J4 | ILMN_9922 |  | polymerase (RNA) II (DNA directed) polypeptide J, 13.3kDa pseudogene. |
| PORCN | ILMN_19819 | X | porcupine homolog (Drosophila) (PORCN), transcript variant A. |
| PPAP2B | ILMN_5681 | 1 | phosphatidic acid phosphatase type 2B (PPAP2B). |
| PPARA | ILMN_7270 | 22 | peroxisome proliferator-activated receptor alpha (PPARA), transcript variant 3. |
| PPARG | ILMN_22381 | 3 | peroxisome proliferator-activated receptor gamma (PPARG). |
| PPFIBP1 | ILMN_21261 | 12 | PTPRF interacting protein, binding protein 1 (liprin beta 1) (PPFIBP1). |
| PPM1A | ILMN_10552 | 14 | protein phosphatase 1A (formerly 2C), magnesium-dependen. |
| PPP1R14A | ILMN_20916 | 19 | protein phosphatase 1, regulatory (inhibitor) subunit 14A (PPP1R14A). |
| PPP1R1C | ILMN_36531 | 2 | protein phosphatase 1, regulatory (inhibitor) subunit 1C (PPP1R1C). |
| PPP2R5C | ILMN_18075 | 22 | protein phosphatase 2, regulatory subunit B', gamma isoform (PPP2R5C). |
| PQLC3 | ILMN_138926 | 6 | PQ loop repeat containing 3 (PQLC3). |
| PRKAG2 | ILMN_671 | 7 | protein kinase, AMP-activated, gamma 2 non-catalytic subunit. |
| PRNPIP | ILMN_38371 |  | PREDICTED: prion protein interacting protein, transcript variant 4 (PRNPIP). |
| PRR11 | ILMN_137089 | 20 | proline rich 11 (PRR11). |
| PRSS22 | ILMN_21409 | 16 | protease, serine, 22 (PRSS22). |
| PTCH1 | ILMN_18640 | 9 | patched homolog 1 (Drosophila) (PTCH1), transcript variant 1c'. |
| PTGS2 | ILMN_29986 | 1 | prostaglandin-endoperoxide synthase 2. |
| PTH2 | ILMN_2034 | 17 | parathyroid hormone 2 (PTH2). |
| PTHLH | ILMN_4025 | 12 | parathyroid hormone-like hormone (PTHLH), transcript variant 3. |
| PTPN14 | ILMN_27079 | 1 | protein tyrosine phosphatase, non-receptor type 14 (PTPN14). |
| PTPRM | ILMN_19957 | 14 | protein tyrosine phosphatase, receptor type, M (PTPRM). |
| RAB11FIP1 | ILMN_4635 | 8 | RAB11 family interacting protein 1 (class I) (RAB11FIP1). |
| RAB11FIP4 | ILMN_427 | 17 | RAB11 family interacting protein 4 (class II) (RAB11FIP4). |
| RAP2B | ILMN_178464 | 3 | RAP2B, member of RAS oncogene family (RAP2B). |
| RAPSN | ILMN_849 | 11 | receptor-associated protein of the synapse (RAPSN). |
| RASSF2 | ILMN_10884 | 20 | Ras association (RalGDS/AF-6) domain family 2 (RASSF2). |
| RASSF6 | ILMN_15686 | 4 | Ras association (RalGDS/AF-6) domain family member 6 (RASSF6). |
| RBM9 | ILMN_13392 | 11 | RNA binding motif protein 9 (RBM9), transcript variant 3. |
| RCAN3 | ILMN_26881 | 1 | RCAN family member 3 (RCAN3). |
| RGS12 | ILMN_19624 | 4 | regulator of G-protein signaling 12 (RGS12). |
| RHOF | ILMN_1762 | 7 | ras homolog gene family, member F (in filopodia) (RHOF). |
| RILP | ILMN_15991 | 17 | Rab interacting lysosomal protein (RILP). |
| RNASE4 | ILMN_16267 | 12 | ribonuclease, RNase A family, 4 (RNASE4), transcript variant 3. |
| RNF128 | ILMN_28439 | X | ring finger protein 128 (RNF128). |
| ROPN1B | ILMN_37645 |  | PREDICTED: ropporin, rhophilin associated protein 1B. |
| RPL28 | ILMN_10642 | 1 | ribosomal protein L28 (RPL28). |
| RPS26P10 | ILMN_40627 | 8 | PREDICTED: ribosomal protein S26 pseudogene 10 (RPS26P10). |
| RRAS | ILMN_23748 |  | related RAS viral (r-ras) oncogene homolog (RRAS). |
| RTN3 | ILMN_20904 | 11 | reticulon 3 (RTN3). |
| RUNX3 | ILMN_16236 | 1 | runt-related transcription factor 3 (RUNX3). |
| SAMD13 | ILMN_10578 | 22 | sterile alpha motif domain containing 13 (SAMD13). |
| SCARNA9 | ILMN_25861 | 19 | small Cajal body-specific RNA 9 (SCARNA9) on chromosome 11, |
| SCEL | ILMN_15617 | 13 | sciellin (SCEL). |
| SCNN1A | ILMN_5697 | 1 | sodium channel, nonvoltage-gated 1 alpha (SCNN1A). |
| SDCCAG1 | ILMN_3136 | 6 | serologically defined colon cancer antigen 1 (SDCCAG1). |
| SDPR | ILMN_11513 | 2 | serum deprivation response (phosphatidylserine binding protein) (SDPR). |
| SENP6 | ILMN_14173 | 6 | SUMO1/sentrin specific peptidase 6 (SENP6). |
| SEPT10 | ILMN_5056 | 2 | septin 10 (SEPT10). |
| SEPT8 | ILMN_36453 | 5 | septin 8 (SEPT8), transcript variant 4. |
| SERPINB1 | ILMN_10210 | 1 | serpin peptidase inhibitor, clade B (ovalbumin), member 1 (SERPINB1). |
| SH2D1A | ILMN_9529 | X | SH2 domain protein 1A, Duncan's disease (lymphoproliferative syndrome). |
| SHD | ILMN_13682 | 19 | Src homology 2 domain containing transforming protein D (SHD). |
| SHFM1 | ILMN_26583 | 12 | split hand/foot malformation (ectrodactyly) type 1 (SHFM1). |
| SLC22A18 | ILMN_20563 | 11 | solute carrier family 22 (organic cation transporter), member 18 (SLC22A18). |
| SLC22A9 | ILMN_172906 | 11 | solute carrier family 22 (organic anion transporter), member 9 (SLC22A9). |
| SLC25A16 | ILMN_165967 | 10 | solute carrier family 25 (mitochondrial carrier; Graves disease autoantigen). |
| SLC2A12 | ILMN_19964 | 6 | solute carrier family 2 (facilitated glucose transporter), member 12 (SLC2A12). |
| SLC2A12 | ILMN_19964 | 1 | solute carrier family 2 (facilitated glucose transporter), member 12 (SLC2A12). |
| SLC2A3 | ILMN_15812 | 11 | solute carrier family 2 (facilitated glucose transporter), member 3 (SLC2A3). |
| SLC30A5 | ILMN_24834 | 5 | solute carrier family 30 (zinc transporter), member 5 (SLC30A5). |
| SLC35F5 | ILMN_22210 | 2 | solute carrier family 35, member F5 (SLC35F5). |
| SMG1 | ILMN_9076 | 16 | PI-3-kinase-related kinase SMG-1 (SMG1). |
| SMG7 | ILMN_29503 | 1 | Smg-7 homolog, nonsense mediated decay factor (C. elegans) (SMG7). |
| SMYD5 | ILMN_26626 | 2 | SMYD family member 5 (SMYD5). |
| SNORD73A | ILMN_669 | 4 | small nucleolar RNA, C/D box 73A (SNORD73A) on chromosome 4. |
| SOCS3 | ILMN_167297 | 17 | suppressor of cytokine signaling 3 (SOCS3). |
| SP8 | ILMN_24886 | 7 | Sp8 transcription factor (SP8). |
| SPAG1 | ILMN_12212 | 8 | sperm associated antigen 1 (SPAG1). |
| SPDYC | ILMN_1637 | 11 | speedy homolog C (Drosophila) (SPDYC). |
| SPOCD1 | ILMN_6339 | 1 | SPOC domain containing 1 (SPOCD1). |
| SPPL3 | ILMN_30223 | 12 | signal peptide peptidase 3 (SPPL3). |
| SRC | ILMN_6831 | 20 | v-src sarcoma (Schmidt-Ruppin A-2) viral oncogene homolog (avian) (SRC). |
| ST8SIA3 | ILMN_16138 | 18 | ST8 alpha-N-acetyl-neuraminide alpha-2,8-sialyltransferase 3 (ST8SIA3). |
| STAG2 | ILMN_14082 | X | stromal antigen 2 (STAG2), transcript variant 4. |
| STAP2 | ILMN_136955 | 19 | signal transducing adaptor family member 2 (STAP2). |
| STEAP1 | ILMN_139257 |  | PREDICTED: six transmembrane epithelial antigen of the prostate 1 (STEAP1). |
| STEAP2 | ILMN_18795 | 7 | six transmembrane epithelial antigen of the prostate 2 (STEAP2). |
| STK40 | ILMN_25410 | 1 | serine/threonine kinase 40 (STK40). |
| SUMO1 | ILMN_14949 | 2 | SMT3 suppressor of mif two 3 homolog 1 (S. cerevisiae) (SUMO1). |
| SUZ12P | ILMN_38242 | 17 | PREDICTED: suppressor of zeste 12 homolog pseudogene. |
| TACSTD2 | ILMN_4004 | 1 | tumor-associated calcium signal transducer 2 (TACSTD2). |
| TAF1C | ILMN_4122 | 16 | TATA box binding protein (TBP)-associated factor, RNA polymerase I, C. |
| TALDO1 | ILMN_138767 |  | PREDICTED: transaldolase 1 (TALDO1). |
| TCEA3 | ILMN_27218 | 1 | transcription elongation factor A (SII), 3 (TCEA3). |
| TCOF1 | ILMN_18418 | 5 | Treacher Collins-Franceschetti syndrome 1 (TCOF1). |
| TCP11L2 | ILMN_21615 | 12 | t-complex 11 (mouse)-like 2 (TCP11L2). |
| TFPI | ILMN_1429 | 7 | tissue factor pathway inhibitor (lipoprotein-associated coagulation inhibitor). |
| TGM2 | ILMN_8134 | 20 | transglutaminase 2 (C polypeptide, protein-glutamine-gamma-glutamyltransferase). |
| TH | ILMN_138010 | 11 | tyrosine hydroxylase (TH), transcript variant 3. |
| TIAM2 | ILMN_9891 | 6 | T-cell lymphoma invasion and metastasis 2 (TIAM2). |
| TICAM2 | ILMN_24482 |  | toll-like receptor adaptor molecule 2 (TICAM2). |
| TIMM17A | ILMN_16007 | 1 | translocase of inner mitochondrial membrane 17 homolog A (yeast). |
| TM4SF18 | ILMN_5533 | 18 | transmembrane 4 L six family member 18 (TM4SF18). |
| TMEM117 | ILMN_21932 | 12 | transmembrane protein 117 (TMEM117). |
| TMEM207 | ILMN_2922 | 3 | transmembrane protein 207 (TMEM207). |
| TMEM45A | ILMN_30168 | 19 | transmembrane protein 45A (TMEM45A). |
| TMEM64 | ILMN_3155 | 8 | transmembrane protein 64 (TMEM64). |
| TMEM87B | ILMN_20699 | 2 | transmembrane protein 87B (TMEM87B). |
| TNFRSF6B | ILMN_14212 | 19 | tumor necrosis factor receptor superfamily, member 6b, decoy. |
| TOMM40L | ILMN_42128 | 1 | translocase of outer mitochondrial membrane 40 homolog (yeast)-like. |
| TRIM16 | ILMN_139304 |  | PREDICTED: tripartite motif-containing 16 (TRIM16). |
| TSC22D3 | ILMN_9893 |  | TSC22 domain family, member 3 (TSC22D3), transcript variant 2. |
| TSGA10 | ILMN_16441 | 2 | testis specific, 10 (TSGA10). |
| TSPAN1 | ILMN_7052 | 1 | tetraspanin 1 (TSPAN1). |
| TTC35 | ILMN_16778 | 8 | tetratricopeptide repeat domain 35 (TTC35). |
| TTC9C | ILMN_5250 | 3 | tetratricopeptide repeat domain 9C (TTC9C). |
| TUBD1 | ILMN_16764 | 17 | tubulin, delta 1 (TUBD1). |
| UBE1C | ILMN_22726 | 3 | ubiquitin-activating enzyme E1C (UBA3 homolog, yeast) (UBE1C). |
| UBE2V1 | ILMN_12143 | 20 | ubiquitin-conjugating enzyme E2 variant 1 (UBE2V1), transcript variant 3. |
| UPK3B | ILMN_24270 | 7 | uroplakin 3B (UPK3B). |
| USP30 | ILMN_5598 | 12 | ubiquitin specific peptidase 30 (USP30). |
| USP54 | ILMN_18622 | 10 | ubiquitin specific peptidase 54 (USP54). |
| VAC14 | ILMN_30132 |  | PREDICTED: Vac14 homolog (S. cerevisiae) (VAC14). |
| VAMP3 | ILMN_19403 | 3 | vesicle-associated membrane protein 3 (cellubrevin) (VAMP3). |
| VEGFA | ILMN_5181 | 6 | vascular endothelial growth factor A (VEGFA), transcript variant 3. |
| VGF | ILMN_9112 | 2 | VGF nerve growth factor inducible (VGF). |
| VIM | ILMN_676 | 1 | vimentin (VIM). |
| WHSC1 | ILMN_27418 | 4 | Wolf-Hirschhorn syndrome candidate 1 (WHSC1), transcript variant 8. |
| YAP1 | ILMN_19290 | 20 | Yes-associated protein 1, 65kDa (YAP1). |
| ZBTB44 | ILMN_30202 | 11 | zinc finger and BTB domain containing 44 (ZBTB44). |
| ZC3H14 | ILMN_22941 | 14 | zinc finger CCCH-type containing 14 (ZC3H14). |
| ZDHHC11 | ILMN_138235 | 5 | zinc finger, DHHC-type containing 11 (ZDHHC11). |
| ZFP36 | ILMN_1557 |  | zinc finger protein 36, C3H type, homolog (mouse) (ZFP36). |
| ZKSCAN5 | ILMN_27210 | 7 | zinc finger with KRAB and SCAN domains 5 (ZKSCAN5). |
| ZNF197 | ILMN_1508 | 3 | zinc finger protein 197 (ZNF197). |
| ZNF566 | ILMN_16016 | 19 | zinc finger protein 566 (ZNF566). |
| ZNF773 | ILMN_1981 | 19 | zinc finger protein 773 (ZNF773). |
| ZSCAN1 | ILMN_19352 | 19 | zinc finger and SCAN domain containing 1 (ZSCAN1). |

B. Genes upregulated after ASH2L silencing

| SYMBOL | ILMN_GENE | CHROMOSOME | DEFINITION |
| --- | --- | --- | --- |
| ACP2 | ILMN_3044 | 11 | acid phosphatase 2, lysosomal (ACP2). |
| AKAP8 | ILMN_10271 | 19 | A kinase (PRKA) anchor protein 8 (AKAP8). |
| ALCAM | ILMN_21054 | 3 | activated leukocyte cell adhesion molecule (ALCAM). |
| AQR | ILMN_166517 | 15 | aquarius homolog (mouse) (AQR), mRNA. |
| AXIN2 | ILMN_26857 | 17 | axin 2 (conductin, axil) (AXIN2). |
| B3GNT2 | ILMN_138549 | 2 | UDP-GlcNAc:betaGal beta-1,3-N-acetylglucosaminyltransferase 2 (B3GNT2). |
| BCLAF1 | ILMN_3336 | 6 | BCL2-associated transcription factor 1 (BCLAF1), transcript variant 2. |
| BRPF1 | ILMN_17423 | 3 | bromodomain and PHD finger containing, 1 (BRPF1), transcript variant 1. |
| C14orf173 | ILMN_41230 | 14 | chromosome 14 open reading frame 173 (C14orf173), transcript variant 2, mRNA. |
| C18orf32 | ILMN_26126 | 18 | chromosome 18 open reading frame 32 (C18orf32), mRNA. |
| CAND1 | ILMN_22065 | 12 | cullin-associated and neddylation-dissociated 1 (CAND1). |
| CASC3 | ILMN_28416 | 17 | cancer susceptibility candidate 3 (CASC3). |
| CCDC86 | ILMN_27103 | 11 | coiled-coil domain containing 86 (CCDC86). |
| CEBPA | ILMN_27029 | 19 | CCAAT/enhancer binding protein (C/EBP), alpha (CEBPA). |
| CENTD3 | ILMN_5090 | 5 | centaurin, delta 3 (CENTD3). |
| CEP72 | ILMN_10995 | 5 | centrosomal protein 72kDa (CEP72), mRNA. |
| CHAC2 | ILMN_1763 | 2 | ChaC, cation transport regulator homolog 2 (E. coli) (CHAC2), mRNA. |
| CHPF | ILMN_22953 | 2 | chondroitin polymerizing factor (CHPF). |
| CHTF18 | ILMN_28360 | 16 | CTF18, chromosome transmission fidelity factor 18 homolog (S, cerevisiae) (CHTF18) |
| CLCN7 | ILMN_8600 | 16 | chloride channel 7 (CLCN7). |
| CNTNAP1 | ILMN_6876 | 17 | contactin associated protein 1 (CNTNAP1), mRNA. |
| COL6A1 | ILMN_138363 | 21 | collagen, type VI, alpha 1 (COL6A1). |
| CPLX1 | ILMN_30247 | 4 | complexin 1 (CPLX1). |
| CPS1 | ILMN_15726 | 2 | carbamoyl-phosphate synthetase 1, mitochondrial (CPS1). |
| CTPS | ILMN_18906 | 1 | CTP synthase (CTPS). |
| CYB5R4 | ILMN_137297 | 6 | cytochrome b5 reductase 4 (CYB5R4). |
| CYP2D6 | ILMN_27062 | 22 | cytochrome P450, family 2, subfamily D, polypeptide 6 (CYP2D6), transcript variant 2 |
| DDX3X | ILMN_139078 | X | DEAD (Asp-Glu-Ala-Asp) box polypeptide 3, X-linked (DDX3X), mRNA. |
| DDX3X | ILMN_139078 | X | DEAD (Asp-Glu-Ala-Asp) box polypeptide 3, X-linked (DDX3X). |
| DEGS1 | ILMN_6374 | 1 | degenerative spermatocyte homolog 1, lipid desaturase (Drosophila) (DEGS1), |
| DMAP1 | ILMN_138222 | 1 | DNA methyltransferase 1 associated protein 1 (DMAP1), transcript variant 2. |
| DNMT1 | ILMN_17904 | 19 | DNA (cytosine-5-)-methyltransferase 1 (DNMT1). |
| DUS3L | ILMN_3805 | 19 | dihydrouridine synthase 3-like (S, cerevisiae) (DUS3L). |
| DUSP4 | ILMN_181455 | 8 | dual specificity phosphatase 4 (DUSP4), transcript variant 2, mRNA. |
| E2F6 | ILMN_14185 | 2 | E2F transcription factor 6 (E2F6), mRNA. |
| EFNB2 | ILMN_3827 | 13 | ephrin-B2 (EFNB2). |
| EIF4G1 | ILMN_5831 | 3 | eukaryotic translation initiation factor 4 gamma, 1 (EIF4G1), transcript variant 1, mRNA. |
| EVPL | ILMN_1544 | 17 | envoplakin (EVPL). |
| FASTKD5 | ILMN_10466 | 20 | FAST kinase domains 5 (FASTKD5). |
| FGF11 | ILMN_8195 | 17 | fibroblast growth factor 11 (FGF11), mRNA. |
| FGF19 | ILMN_18897 | 11 | fibroblast growth factor 19 (FGF19). |
| FGF9 | ILMN_1771 | 13 | fibroblast growth factor 9 (glia-activating factor) (FGF9). |
| FGFR3 | ILMN_22960 | 4 | fibroblast growth factor receptor 3 (achondroplasia, thanatophoric dwarfism) (FGFR3), |
| FKBP5 | ILMN_16562 | 6 | FK506 binding protein 5 (FKBP5). |
| FOXC1 | ILMN_23624 | 6 | forkhead box C1 (FOXC1). |
| FOXF1 | ILMN_11804 | 16 | forkhead box F1 (FOXF1), mRNA. |
| FOXO3 | ILMN_15283 | 6 | forkhead box O3 (FOXO3), transcript variant 1. |
| GCC1 | ILMN_21168 | 7 | GRIP and coiled-coil domain containing 1 (GCC1), |
| GGA2 | ILMN_17168 | 16 | golgi associated, gamma adaptin ear containing, ARF binding protein 2 (GGA2), |
| GNB1 | ILMN_26098 | 1 | guanine nucleotide binding protein (G protein), beta polypeptide 1 (GNB1), |
| GNS | ILMN_6937 | 12 | glucosamine (N-acetyl)-6-sulfatase (Sanfilippo disease IIID) (GNS), |
| HDAC2 | ILMN_28766 | 6 | histone deacetylase 2 (HDAC2), |
| HYOU1 | ILMN_659 | 11 | hypoxia up-regulated 1 (HYOU1), |
| ICMT | ILMN_137648 | 1 | isoprenylcysteine carboxyl methyltransferase (ICMT), |
| IDH3A | ILMN_3303 | 15 | isocitrate dehydrogenase 3 (NAD+) alpha (IDH3A), |
| INPP5E | ILMN_11866 | 9 | inositol polyphosphate-5-phosphatase, 72 kDa (INPP5E), |
| ISG20L1 | ILMN_12401 | 15 | interferon stimulated exonuclease gene 20kDa-like 1 (ISG20L1), |
| KCNH3 | ILMN_11930 | 12 | potassium voltage-gated channel, subfamily H (eag-related), member 3 (KCNH3), |
| KIAA0913 | ILMN_16257 | 10 | KIAA0913 (KIAA0913), |
| KIAA1712 | ILMN_5346 | 4 | KIAA1712 (KIAA1712). |
| KIAA1737 | ILMN_24671 | 14 | KIAA1737 (KIAA1737), |
| KLHDC5 | ILMN_1962 | 12 | kelch domain containing 5 (KLHDC5), |
| KTELC1 | ILMN_15488 | 3 | KTEL (Lys-Tyr-Glu-Leu) containing 1 (KTELC1), |
| KTI12 | ILMN_27125 | 1 | KTI12 homolog, chromatin associated (S, cerevisiae) (KTI12), |
| LCOR | ILMN_173510 | 10 | ligand dependent nuclear receptor corepressor (LCOR), |
| LEP | ILMN_10827 | 7 | leptin (obesity homolog, mouse) (LEP). |
| LMF2 | ILMN_11132 | 22 | lipase maturation factor 2 (LMF2), |
| LMNB2 | ILMN_24712 | 19 | lamin B2 (LMNB2), |
| LPCAT1 | ILMN_15076 | 5 | lysophosphatidylcholine acyltransferase 1 (LPCAT1), |
| LRRC14 | ILMN_29237 | 8 | leucine rich repeat containing 14 (LRRC14), |
| LSM14A | ILMN_19241 | 19 | LSM14A, SCD6 homolog A (S, cerevisiae) (LSM14A), |
| LYRM2 | ILMN_30197 | 6 | LYR motif containing 2 (LYRM2), |
| LZTR1 | ILMN_18977 | 22 | leucine-zipper-like transcription regulator 1 (LZTR1), |
| MANEAL | ILMN_11393 | 1 | mannosidase, endo-alpha-like (MANEAL), transcript variant 1. |
| MAP6D1 | ILMN_7455 | 3 | MAP6 domain containing 1 (MAP6D1), |
| MED22 | ILMN_22202 | 9 | mediator complex subunit 22 (MED22), transcript variant c, |
| MEPCE | ILMN_8017 | 7 | methylphosphate capping enzyme (MEPCE), |
| MGEA5 | ILMN_11399 | 10 | meningioma expressed antigen 5 (hyaluronidase) (MGEA5), |
| MKX | ILMN_28370 | 10 | mohawk homeobox (MKX), |
| MLSTD2 | ILMN_11547 | 11 | male sterility domain containing 2 (MLSTD2), |
| MRPS18C | ILMN_12033 | 4 | mitochondrial ribosomal protein S18C (MRPS18C). |
| MSX1 | ILMN_137891 | 4 | msh homeobox 1 (MSX1), |
| MTCH2 | ILMN_2631 | 11 | mitochondrial carrier homolog 2 (C. elegans) (MTCH2). |
| NADK | ILMN_29863 | 1 | NAD kinase (NADK), |
| NCOA5 | ILMN_3004 | 20 | nuclear receptor coactivator 5 (NCOA5). |
| NLRP2 | ILMN_17259 | 19 | NLR family, pyrin domain containing 2 (NLRP2), |
| NOL5A | ILMN_13841 | 20 | nucleolar protein 5A (56kDa with KKE/D repeat) (NOL5A), |
| NR6A1 | ILMN_2143 | 9 | nuclear receptor subfamily 6, group A, member 1 (NR6A1), transcript variant 2. |
| NRBP2 | ILMN_27830 | 8 | nuclear receptor binding protein 2 (NRBP2), |
| NT5DC2 | ILMN_20328 | 3 | 5'-nucleotidase domain containing 2 (NT5DC2), |
| NUDT16L1 | ILMN_20270 | 16 | nudix (nucleoside diphosphate linked moiety X)-type motif 16-like 1 (NUDT16L1). |
| NXF1 | ILMN_11773 | 11 | nuclear RNA export factor 1 (NXF1), transcript variant 1, |
| OGDHL | ILMN_15789 | 10 | oxoglutarate dehydrogenase-like (OGDHL), |
| PAQR3 | ILMN_20371 | 4 | progestin and adipoQ receptor family member III (PAQR3), |
| PFAS | ILMN_17615 | 17 | phosphoribosylformylglycinamidine synthase (FGAR amidotransferase) (PFAS), |
| POLR3B | ILMN_11759 | 12 | polymerase (RNA) III (DNA directed) polypeptide B (POLR3B), |
| PPAP2A | ILMN_5193 | 5 | phosphatidic acid phosphatase type 2A (PPAP2A), transcript variant 2. |
| PPP4R1 | ILMN_6519 | 18 | protein phosphatase 4, regulatory subunit 1 (PPP4R1), transcript variant 2, |
| PPRC1 | ILMN_10445 | 10 | peroxisome proliferator-activated receptor gamma, coactivator-related 1 (PPRC1), |
| PRELID1 | ILMN_28597 | 5 | PRELI domain containing 1 (PRELID1), |
| PSMA7 | ILMN_13260 | 20 | proteasome (prosome, macropain) subunit, alpha type, 7 (PSMA7), transcript variant 2. |
| PTDSS1 | ILMN_4241 | 8 | phosphatidylserine synthase 1 (PTDSS1), |
| PTK7 | ILMN_11421 | 6 | PTK7 protein tyrosine kinase 7 (PTK7), transcript variant PTK7-2, |
| PYGB | ILMN_21544 | 20 | phosphorylase, glycogen; brain (PYGB), |
| RASSF1 | ILMN_11841 | 3 | Ras association (RalGDS/AF-6) domain family 1 (RASSF1), transcript variant B. |
| RBM12 | ILMN_22052 | 20 | RNA binding motif protein 12 (RBM12), transcript variant 1, |
| RBM14 | ILMN_16867 | 11 | RNA binding motif protein 14 (RBM14), |
| RBM16 | ILMN_3973 | 6 | RNA binding motif protein 16 (RBM16), |
| RBM38 | ILMN_20092 | 20 | RNA binding motif protein 38 (RBM38), transcript variant 2, |
| RCBTB2 | ILMN_15980 | 13 | regulator of chromosome condensation (RCC1) and BTB (POZ) |
| RIC8B | ILMN_3462 | 12 | resistance to inhibitors of cholinesterase 8 homolog B (C. elegans) (RIC8B). |
| RIMS3 | ILMN_21581 | 1 | regulating synaptic membrane exocytosis 3 (RIMS3), |
| RNF145 | ILMN_27136 | 5 | ring finger protein 145 (RNF145), |
| RNF19A | ILMN_10959 | 8 | ring finger protein 19A (RNF19A), transcript variant 2. |
| RNF219 | ILMN_38012 | 13 | ring finger protein 219 (RNF219), |
| RRP1B | ILMN_14288 | 21 | ribosomal RNA processing 1 homolog B (S, cerevisiae) (RRP1B), |
| RRS1 | ILMN_9627 | 8 | RRS1 ribosome biogenesis regulator homolog (S, cerevisiae) (RRS1), |
| SAR1B | ILMN_16595 | 5 | SAR1 gene homolog B (S, cerevisiae) (SAR1B), transcript variant 1, |
| SCRN2 | ILMN_2736 | 17 | secernin 2 (SCRN2). |
| SETMAR | ILMN_17510 | 3 | SET domain and mariner transposase fusion gene (SETMAR), |
| SF4 | ILMN_9581 | 19 | splicing factor 4 (SF4), transcript variant c, |
| SFPQ | ILMN_5703 | 1 | splicing factor proline/glutamine-rich(polypyrimidine tract binding protein associated). |
| SFRS6 | ILMN_24964 | 20 | splicing factor, arginine/serine-rich 6 (SFRS6), |
| SFRS7 | ILMN_7620 | 2 | splicing factor, arginine/serine-rich 7, 35kDa (SFRS7), |
| SH3GLB2 | ILMN_14480 | 9 | SH3-domain GRB2-like endophilin B2 (SH3GLB2), |
| SHRM | ILMN_16821 | 4 | shroom (SHRM), |
| SIGIRR | ILMN_18194 | 11 | single immunoglobulin and toll-interleukin 1 receptor (TIR) domain (SIGIRR). |
| SLC25A10 | ILMN_24086 | 17 | solute carrier family 25 (mitochondrial carrier; dicarboxylate transporter). |
| SLC41A1 | ILMN_2825 | 1 | solute carrier family 41, member 1 (SLC41A1), |
| SLC4A2 | ILMN_6956 | 7 | solute carrier family 4, anion exchanger, member 2 |
| SNAPC4 | ILMN_3646 | 9 | small nuclear RNA activating complex, polypeptide 4, 190kDa (SNAPC4), |
| SNORD13 | ILMN_135987 | 8 | small nucleolar RNA, C/D box 13 (SNORD13) on chromosome 8, |
| SPEN | ILMN_3876 | 1 | spen homolog, transcriptional regulator (Drosophila) (SPEN), |
| SRPRB | ILMN_2452 | 3 | signal recognition particle receptor, B subunit (SRPRB), |
| STC2 | ILMN_28725 | 5 | stanniocalcin 2 (STC2), |
| STK35 | ILMN_27051 | 20 | serine/threonine kinase 35 (STK35), |
| TEAD2 | ILMN_4452 | 19 | TEA domain family member 2 (TEAD2), |
| TEX2 | ILMN_27579 | 17 | testis expressed 2 (TEX2), |
| TLN1 | ILMN_18029 | 9 | talin 1 (TLN1), |
| TLR5 | ILMN_18399 | 1 | toll-like receptor 5 (TLR5). |
| TMEM132A | ILMN_41781 | 11 | transmembrane protein 132A (TMEM132A), transcript variant 2, |
| TMEM177 | ILMN_22670 | 2 | transmembrane protein 177 (TMEM177), |
| TMEM184B | ILMN_10219 | 22 | transmembrane protein 184B (TMEM184B), |
| TMEM200A | ILMN_19059 | 6 | transmembrane protein 200A (TMEM200A), |
| TMEM41A | ILMN_28063 | 3 | transmembrane protein 41A (TMEM41A), |
| TMEM50B | ILMN_138521 | 21 | transmembrane protein 50B (TMEM50B), |
| TMEM63A | ILMN_932 | 1 | transmembrane protein 63A (TMEM63A). |
| TOP1MT | ILMN_15321 |  | PREDICTED: topoisomerase (DNA) I, mitochondrial (TOP1MT), |
| TRRAP | ILMN_18258 | 7 | transformation/transcription domain-associated protein (TRRAP), |
| TRSPAP1 | ILMN_23632 | 1 | tRNA selenocysteine associated protein 1 (TRSPAP1), transcript variant 2. |
| TSEN2 | ILMN_2707 | 3 | tRNA splicing endonuclease 2 homolog (S, cerevisiae) (TSEN2), |
| TSPAN17 | ILMN_18131 | 5 | tetraspanin 17 (TSPAN17), transcript variant 1, |
| TTC15 | ILMN_21565 | 2 | tetratricopeptide repeat domain 15 (TTC15), |
| TTL | ILMN_14027 | 2 | tubulin tyrosine ligase (TTL), |
| TUBB2A | TUBB | 6 | tubulin, beta 2A (TUBB2A), |
| TUBB4 | ILMN_23388 | 19 | tubulin, beta 4 (TUBB4), |
| TXLNA | ILMN_1892 | 1 | taxilin alpha (TXLNA), |
| TYK2 | ILMN_29800 | 19 | tyrosine kinase 2 (TYK2), |
| UBE2G2 | ILMN_22176 | 21 | ubiquitin-conjugating enzyme E2G 2 (UBC7 homolog, yeast), transcript variant 1, |
| UBE2I | ILMN_27920 | 16 | ubiquitin-conjugating enzyme E2I (UBC9 homolog, yeast), transcript variant 4. |
| UBP1 | ILMN_25221 | 3 | upstream binding protein 1 (LBP-1a) (UBP1), |
| USP38 | ILMN_28204 | 4 | ubiquitin specific peptidase 38 (USP38), |
| VASN | ILMN_138591 | 16 | vasorin (VASN), |
| YWHAH | ILMN_27184 | 22 | tyrosine 3-monooxygenase/tryptophan 5-monooxygenase activation protein. |
| ZBED1 | ILMN_14030 | X | zinc finger, BED-type containing 1 (ZBED1), |
| ZBED4 | ILMN_8641 | 22 | zinc finger, BED-type containing 4 (ZBED4), |
| ZBED5 | ILMN_17879 | 11 | zinc finger, BED-type containing 5 (ZBED5), |
| ZFP36L2 | ILMN_169693 | 2 | zinc finger protein 36, C3H type-like 2 (ZFP36L2), |
| ZNF342 | ILMN_7933 | 19 | zinc finger protein 342 (ZNF342), |
| ZNF35 | ILMN_15315 | 3 | zinc finger protein 35 (ZNF35), |
| ZNF512 | ILMN_5859 | 2 | zinc finger protein 512 (ZNF512), |
| ZNF518B | ILMN_3143 | 4 | zinc finger protein 518B (ZNF518B), |
| ZNF559 | ILMN_11090 | 19 | zinc finger protein 559 (ZNF559), |
|  |  |  |  |
| ZNF696 | ILMN_21311 | 8 | zinc finger protein 696 (ZNF696), |
