# Supplementary material for: NF-Y Recruits Ash2L to Impart H3K4 Trimethylation on CCAAT Promoters
Source: PLoS One. 2011 Mar 21;6(3):e17220. doi: 10.1371/journal.pone.0017220 (PMC3061855; doi:10.1371/journal.pone.0017220)
Supplement: Figure S2 — Transcription Factor Binding Site (TFBS) analysis of the promoters (-500/+100 from the Transcriptional Start Site) of the Ash2L-regulated genes derived from the profiling experiments. (DOC) [file pone.0017220.s002.doc]

| **TF_NAME** | LOGO | MATRIX_ID | Z_SCORE | P_VALUE |
| --- | --- | --- | --- | --- |
| **NF-YA** |  | Dolfini et al. Ref. 18 | 2.65185 | 0.00389626 |
| **Klf4** | 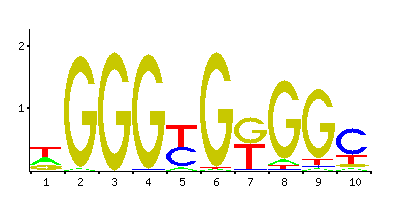 | MA0039.2 | 2.24908 | 0.0120964 |
| **Stat3** | 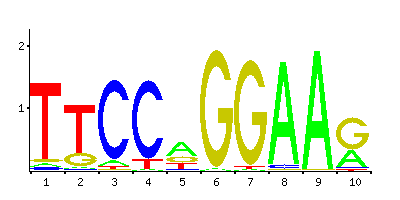<INPUT VALUE=\ | MA0144.1 | 2.07726 | 0.0186067 |
| **Pax2** | 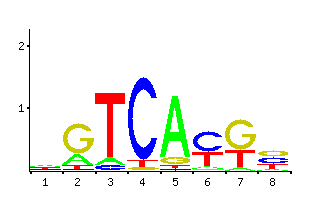 | MA0067.1 | 2.07831 | 0.0186399 |
| **TBP** | 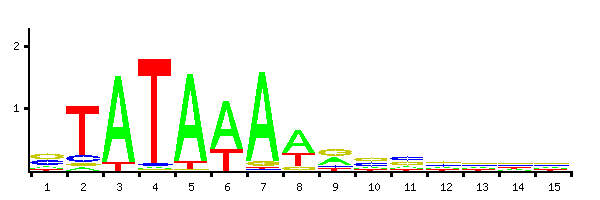 | MA0108.2 | 1.93156 | 0.0264203 |
| **Myf** | 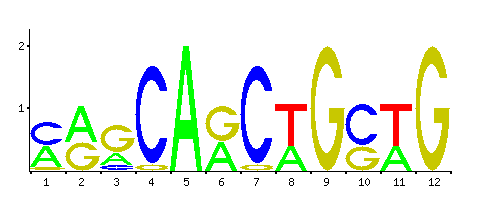 | MA0055.1 | 1.78919 | 0.0364266 |
| **MIZF** | 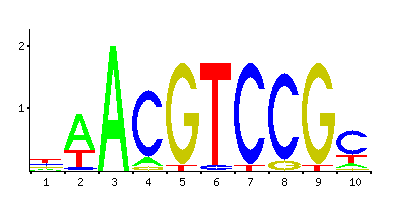 | MA0131.1 | 1.6672 | 0.0474185 |
| **Sox17** | 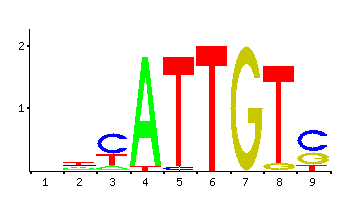 | MA0078.1 | 1.55573 | 0.0595298 |

Figure S2 Promoters of genes downregoleted after ASH2 silencing, were analyzed with Pscan alghortim (Zambelli F. et al.Nucleic Acids Res. 2009 Jul 1;37) using the matrices of the JASPAR database.
