# Supplementary material for: NF-Y Recruits Ash2L to Impart H3K4 Trimethylation on CCAAT Promoters
Source: PLoS One. 2011 Mar 21;6(3):e17220. doi: 10.1371/journal.pone.0017220 (PMC3061855; doi:10.1371/journal.pone.0017220)
Supplement: Figure S3 — List of primers used in q-RT-PCR and ChIPs. (PDF) [file pone.0017220.s003.pdf]

ChIP-PCR PRIMERS	
MDM2	GGTTGACTCAGCTTTTCCTCTTG
MDM2	GGAAAATGCATGGTTTAAATAGCC
UNG	CGCGCGCCTATAATCCTAGC
UNG	GCTTGATGGCTCACGTCTGT
NCOA5	GTGGTCCGGAGGTTACAGGAC
NCOA5	GAGCACATTCCCTCCTCCCTA
TICAM2	AGCGCTCTACCACCAACGATT
TICAM2	GACAAGAGTCTCGCCCTGTCA
HDAC1	CCTCTCCGGGCTGCCCTTG
HDAC1	CCTCCTGGGCTCACCTATAGC
ERP70	CTCACGTTAGGGCTCGGAGTTT
ERP70	GGAAAAACCCACGGAAGTCGT
CHOP	CTCGTGACCCAAAGCCACTTC
CHOP	GGACCCCAAAACCTACCAATCAG
CCN B1	TGTCACCTTCCAAAGGCCACTA
CCN B1	AGAAGAGCCAGCCTAGCCTCAG
CCN B2	AGAGGCGTCCTACGTCTGCTTT
CCN B2	ATTCAAATACCGCGTCGCTTG
HSPA5	AGGGGAGGACCTGAACGGTTAC
HSPA5	TGTTGTCTCGGCCAGTATCGAG
PCNA	GAGTCAAAGAGGCGGGGAGAC
PCNA	CTTGCGGGGAAGACTTTAGGG
CKS2	CCCCGTGACGTACCTATCTT
CKS2	ACAACTCGCCGGAGACTAAC
HERPUD	ATTGGGCCACGTTGGGAGAGT
HERPUD	CAACGACAGTTCACGTCTCTGG
ID2	TCTTGATAGACGTGCCACCTTCC
ID2	TAACGGACCTCACGGGACTGA
RT-PCR PRIMERS	
ANKRD37	AATCCACATGACCAAGCGAGA
ANKRD37	TAAGTCAGTGGGCGTGAGAGG
TICAM2	AAGAGAAGCTCAAGGCCGAAG
TICAM2	TCCAAGGCAGAAGAGGAAAAC
ALSR2CR2	GCCATCAGCAAGCAGTTTATTG
ALSR2CR2	AAGCAGGAGGCAACAGTGAAA
ID1	TCCGCTCAGCACCCTCAAC
ID1	CGCTTCAGCGACACAAGATG
MDM2	CAGCTTCGGAACAAGAGACC
MDM2	GGCACGCCAAACAAATCTCC
APOBEC3B	CTGCTTCTCCTGGGGCTGT
APOBEC3B	GACATCCCTGGCGGTACAC
DDX50	ATAGCTCAAGCACGGACAGG
DDX50	GCCACGCTGAGTTTCCTAGT
CDKN1A	CTGGAGACTCTCAGGGTCGAA
CDKN1A	GGATTAGGGCTTCCTCTTGGA
CDKN1B	CCACGAAGAGTTAACCCGGG
CDKN1B	GTCTGCTCCACAGAACCGGC
YWHA	ACTTTTGGTACATTGTGGCTTCAA
YWHA	CCGCCAGGACAAACCAGTAT
ID2	CCTCAACACGGATATCAGCA
ID2	AGAACACCCTGGGAAGATGA
CCNA2	TATTGCTGGAGCTGCCTTTC
CCNA2	CTCTGGTGGGTTGAGGAGAG
CBL	GCTGGTTGTCTCTGGATGGT
CBL	CCCCTGACTCATGAGGTTCT
NES	GCAGCAGCTGGCGCACCTCAAGA
NES	GCCAGGTGTTTGCAGCCGGGAGT
DNAJ	GAGTGGAACCCGGAGACATTG
DNAJ	GATACTGCGGCATCCCTTCAC
HERPUD1	CTACTCCTCCCTGAGCAGATT
HERPUD1	GGTTGGGGTCTTAGTTTCAG

CKS2	GGAGTGGAGGAGACTTGGTG
CKS2	CAGCTCATGCACAGGTATGG
CHOP	GCCAAAATCAGAGCTGGAACCT
CHOP	ACAGTGTCCCGAAGGAGAAAGG
CCNB1	CACTTCCTTCGGAGAGCATC
CCNB1	CAGGTGCTGCATAACTGGAA
PCNA	GAGGCTCAATTCTTGGCTCTGC
PCNA	CCGGGTGTTTACCTTCCAGTCA
MBD6	GAGGGTGCTGTGCTCTACATC
MBD6	CCGAAGACTATGGGAAACAGCC
SDHA	TGGGAACAAGAGGGCATCTG
SDHA	CCACCACTGCATCAAATTCATG
ERDJ4	AATAAGAGCCCGGATGCTGAAG
ERDJ4	GCTTCTTGGATCCAGTGTTTTGG
HDAC1	CCGAAGAGGAGAAAACCAAGG
HDAC1	CCAGAGCTGGAGAGGTCCATT
UNG	CTCCTTCTCAACGCTGTCCT
UNG	GAGGGATGAGCCGTCTGTAG
ERP70	AGAGTGGGGAGGATGTCAATG
ERP70	TGACGGGTCCCTTGTTGTTCT
ATF4	TTCCTGAGCAGCGAGGTGTTG
ATF4	TCCAATCTGTCCCGGAGAAGG
TRA1	GGAGAGTCGTGAAGCAGTTG
TRA1	ATACCCTGACCGAAGCGTTG
DOT1	TTTGCCTTTGGTCCTGAGGTG
DOT1	TCAGAGGTGCAAAGGGTTTCG
HSPA5	GGAACACAGTGGTGCCTACCAA
HSPA5	GGAGCAGGAGGAATTCCAGTCA
WDR5	CATCTGGAACCTTCAGACGAAAGA
WDR5	GCAGCAGAGGCGATGTAGTTT
RBbP5	TCAATTGAGTTTGCCCGGAAG
RBbP5	TGCATAGGTTCAGGCTCTCCA
MENIN1	TACCACTGTCGCAACCGCAAT
MENIN1	TTCAGCAGGTTGGGGATGACA
CCNJ	TCTCCAACGTGGCCTACAAGA
CCNJ	GTCCTGCTTGCCCTCTCTGTT
NCOA5	TGCTATTGTCATCACCCAGCAA
NCOA5	CCGGCACTCATTCTTGTAACG
